# Human-induced marine ecological degradation: micropaleontological perspectives

**DOI:** 10.1002/ece3.425

**Published:** 2012-11-15

**Authors:** Moriaki Yasuhara, Gene Hunt, Denise Breitburg, Akira Tsujimoto, Kota Katsuki

**Affiliations:** 1 School of Biological Sciences, University of Hong KongHong Kong SAR, China; 2 Swire Institute of Marine Science, University of Hong KongHong Kong SAR, China; 3 Department of Earth Sciences, University of Hong KongHong Kong SAR, China; 4 Department of Paleobiology, National Museum of Natural History, Smithsonian InstitutionWashington, DC, 20013-7012, USA; 5 Smithsonian Environmental Research CenterEdgewater, Maryland, 21037, USA; 6 Faculty of Education, Shimane UniversityMatsue, Shimane, 690-8504, Japan; 7 Geological Research Division, Korea Institute of Geoscience and Mineral ResourcesDaejeon, 305-350, Korea

**Keywords:** Eutrophication, hypoxia, marine ecosystems, microfossils, pollution, species diversity

## Abstract

We analyzed published downcore microfossil records from 150 studies and reinterpreted them from an ecological degradation perspective to address the following critical but still imperfectly answered questions: (1) How is the timing of human-induced degradation of marine ecosystems different among regions? (2) What are the dominant causes of human-induced marine ecological degradation? (3) How can we better document natural variability and thereby avoid the problem of shifting baselines of comparison as degradation progresses over time? The results indicated that: (1) ecological degradation in marine systems began significantly earlier in Europe and North America (∼1800s) compared with Asia (post-1900) due to earlier industrialization in European and North American countries, (2) ecological degradation accelerated globally in the late 20th century due to post-World War II economic growth, (3) recovery from the degraded state in late 20th century following various restoration efforts and environmental regulations occurred only in limited localities. Although complex in detail, typical signs of ecological degradation were diversity decline, dramatic changes in total abundance, decrease in benthic and/or sensitive species, and increase in planktic, resistant, toxic, and/or introduced species. The predominant cause of degradation detected in these microfossil records was nutrient enrichment and the resulting symptoms of eutrophication, including hypoxia. Other causes also played considerable roles in some areas, including severe metal pollution around mining sites, water acidification by acidic wastewater, and salinity changes from construction of causeways, dikes, and channels, deforestation, and land clearance. Microfossils enable reconstruction of the ecological history of the past 10^2^–10^3^ years or even more, and, in conjunction with statistical modeling approaches using independent proxy records of climate and human-induced environmental changes, future research will enable workers to better address Shifting Baseline Syndrome and separate anthropogenic impacts from background natural variability.

## Introduction

What are the dominant causes and mechanisms of the deterioration of marine organisms, communities, and ecosystems by anthropogenic activities (hereafter marine ecological degradation)? How is timing of marine ecological degradation different among regions and how can we understand changes in such systems in the face of shifting understanding of natural states and variability (Pauly 1995; Knowlton and Jackson 2008)? These critical questions are still answered imperfectly. Marine ecological degradation is a serious concern both because of the changes in marine systems themselves, and because of potential consequences for human populations as ecosystem services historically provided by marine systems are lost (Jackson et al. 2001; Worm et al. 2006; Palumbi et al. 2009). Numerous lines of evidence have shown that marine ecological degradations, for example, biodiversity loss, population collapse, invasion of exotic species, and various faunal/floral changes, are now apparent in most, if not all, marine ecosystems (Lotze et al. 2006; Halpern et al. 2008; Jackson 2008). Causes include increased nutrient loads resulting in zones of low dissolved oxygen and other symptoms of eutrophication (Kemp et al. 2005; Diaz and Rosenberg 2008; Breitburg et al. 2009), fisheries exploitation (Jackson 2001; Jackson et al. 2001; Worm et al. 2006), physical habitat destruction (Jackson 2001; Jackson et al. 2001; Coleman and Williams 2002), ocean acidification (Orr et al. 2005; Hoegh-Guldberg et al. 2007; Fabry et al. 2008), pollution (Shahidul Islam and Tanaka 2004), land clearance/modification (Lotze et al. 2005; Airoldi and Beck 2007; Willard and Cronin 2007), and global warming (Hoegh-Guldberg and Bruno 2010).

Meta-analyses have detailed the general historical process of marine ecological degradation for the last 3000 years using fisheries, ecological, historical, archeological, and paleontological records, and have revealed rapid biodiversity and population loss starting at ∼1800, the onset of industrialization (Lotze et al. 2006; Worm et al. 2006). A similar approach was also used for coral reef ecosystems, suggesting even earlier timing of the inception of ecological degradation (Pandolfi et al. 2003). From these studies, we have a general idea of the current status and the historical process of marine ecological degradation. However, higher temporal resolution analyses of the long-term history of marine ecological degradation are scarce for the past few centuries and millennia, mainly because of the difficulty of obtaining long-term, continuous ecological records. This difficulty is due to a number of factors: (1) biological monitoring usually covers no more than the past 20 years (Borja et al. 2010), which is too short a time period to understand the long-term history of human-caused ecological degradation; (2) fisheries harvest records covering hundreds of years (Lotze and Milewski 2004) are available for only a few commercial fishes and may be artificially and politically biased; and (3) historical and archeological records are often exceedingly fragmentary in time and space. Furthermore, data are usually available only from waters of the coasts of North America, Europe, Australia, and New Zealand (e.g., Worm et al. 2006, 2009).

The fossil record has the potential to provide some essential information that is absent in these other records (Jablonski et al. 2003, 2006). Kidwell has recently shown the utility of comparing live and dead (sub-fossil) molluscan assemblages to detect changes in relative abundance attributable to environmental degradation (Kidwell 2007). Several researchers have also emphasized the importance of a paleoecological approach (Gorham et al. 2001) and integrated it with fisheries, ecological, historical, and archeological records (Jackson et al. 2001; Worm et al. 2006). However, the geological records of most fossil groups lack sufficient time resolution to elucidate a detailed history of marine ecological degradation during past centuries.

Marine microfossils include protists (foraminifera and radiolaria), microscopic algae (diatoms, dinoflagellates, and coccolithophores), and crustaceans (ostracods). These organisms are abundantly and continuously preserved as fossils in cores with potentially continuous sedimentary records, and so provide excellent temporal resolution and long-term ecological data for the past hundreds to thousands of years. Various radiometric dating methods can provide robust chronologies for sediment cores. Furthermore, each group of microfossils is composed of many species with various habitat and ecological preferences and well-known taxonomy. Thus, they are useful proxies or model organisms to infer states of whole marine ecosystems in the past (Thomas and Gooday 1996; Cronin and Raymo 1997; Yasuhara and Cronin 2008; Yasuhara et al. 2008). Although several researchers have shown the importance of microfossils in understanding marine ecological degradation (Tikkanen et al. 1997; Willard and Cronin 2007; Yasuhara et al. 2007; Tsujimoto et al. 2008), many studies focused on microfossil-based reconstruction of pollution history (Sen Gupta et al. 1996; Clarke et al. 2006; Gooday et al. 2009) rather than reconstruction of the biotic response to pollution. This situation is similar to that in paleolimnology. As Smol (2009) pointed out, paleolimnological data can typically be used in two important ways: (1) to use the information preserved in lake sediments to reconstruct past population of specific organisms or groups of organisms that comprise important components of aquatic ecosystems; (2) to use it to reconstruct past environmental conditions such as pH and oxygen contents. Most paleolimnological studies are typically the latter (Smol 2009). The same is true for marine paleoecology. In the present paper, we focus on the former perspective – biotic response, not paleoenvironmental reconstruction – and use the word “paleoecology” only for studies that use the (microfossil) information preserved in marine sediments to reconstruct past population of specific organisms or groups of organisms that comprise important components of marine ecosystems (cf. Smol 2009).

Microfossils can be useful to sort out the relative importance of climate changes and human-induced environmental changes. Periods of ecological degradation overlap with several serious climatic events including the Little Ice Age. With sufficiently detailed records, it may be possible to evaluate the relative importance of climate and human-induced environmental changes on marine ecosystems, including the degree to which human-induced post-20th century global warming has had a significant effect on marine ecosystems.

Numerous lines of evidences suggest regional differences in the timing of the inception of marine ecological degradation. For example, when one compares a review paper based on Asian records (Pauly and Thia-Eng 1988) with those based on North American and European records (Jackson et al. 2001; Worm et al. 2006), a clear difference in timing is apparent, with the degradation of Asian ecosystems occurring later on average. Furthermore, most human-induced environmental problems first appeared in Europe before spreading to other regions (Huettmann 2012), but these qualitative patterns are yet to be tested statistically on a global scale. Previous case studies are largely restricted to developed countries because long-term monitoring and robust fishery, historical, and archeological data are usually available only from North America, Europe, and Australia. Microfossil records can help to broaden this coverage, however, because one well-chosen sediment core allows for the reconstruction of hundred years of ecosystem history, even in the complete absence of local biological monitoring.

Here, we review and analyze published microfossil records from 150 studies and synthesize them in order to understand the spatial and temporal context of ecological degradation. Our objective is to use microfossil records to answer critical questions about marine degradation listed above, and to:

1. examine long-term global trends of marine ecological degradation, focusing on evidence on the timing of inception (i.e., start of degradation), acceleration (i.e., increasing severity of degradation), and ecosystem recovery;
2. provide an overview of the nature of marine ecological degradation evident in microfossil assemblages;
3. review regional trends in marine ecological degradation; and
4. discuss problems, solutions, and future outlook of micropaleontological analyses to investigate marine ecological degradation.

## Methods

**Figure 1:**
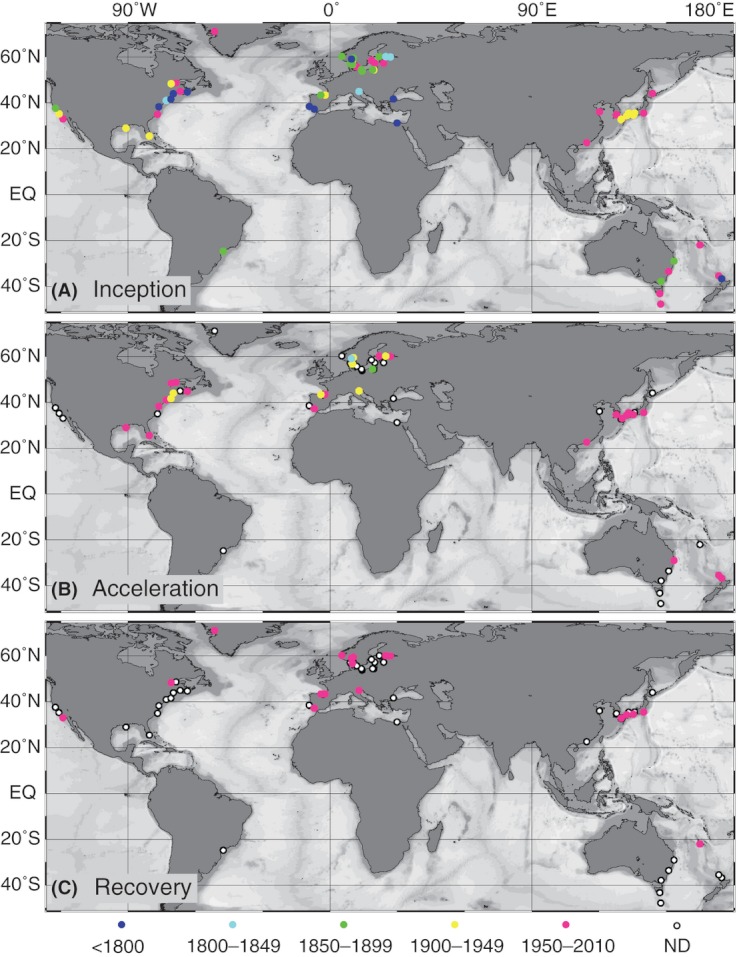
Global distribution of marine ecological degradations in downcore microfossil records. Complete data are found in Table S1. Distribution of (A) inception, (B) acceleration, and (C) recovery ages [five categories of <1800 (blue), 1800–1849 (light blue), 1850–1899 (green), 1900–1949 (yellow), and 1950–2010 (pink)] of marine ecological degradation detected in microfossil records. ND (open circle): not detected.

We compiled marine (in a broad sense, including deep sea, pelagic, shallow marine, estuarine, and brackish-water lake) microfossil records in dated sediment cores (hereafter downcore microfossil records) that preserved evidence of ecological degradation (Figs. 1^5^; Online Table S1, Fig. S1, and Supplementary Text S1; see “Nature of Marine Ecological Degradation: Paleoecological Information from Microfossils” section below). These records were mainly composed of data on foraminifera, diatoms, dinoflagellate cysts, and ostracods (Figs S2–3; Table S1). Diatoms and dinoflagellates include both planktic and benthic species. Although foraminifera and ostracods also have both planktic and benthic species, with the exception of one planktic foraminiferal study on global warming (see Table S1 and *Global warming* section), our database includes only benthic taxa. Planktic foraminifera are rare in shallow, coastal, and/or brackish-water environments where marine ecological degradations are prominent. Planktic ostracods are rarely preserved as fossils because of their weakly calcified carapaces (Schellenberg 2007).

**Figure 2:**
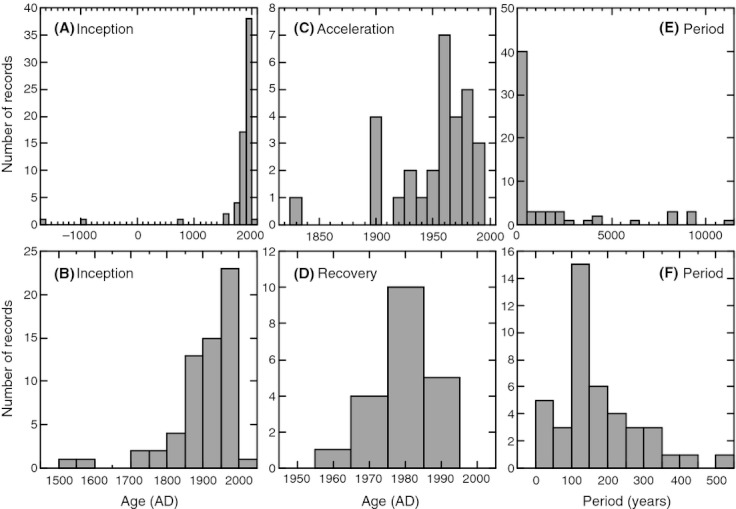
Age distribution of microfossil-based marine ecological degradation records. Histograms showing distribution of (A; B for closeup) inception, (C) acceleration, and (D) recovery ages of marine ecological degradations and of (E; F for closeup) covering period of microfossil records. Data from Table S1.

**Figure 3:**
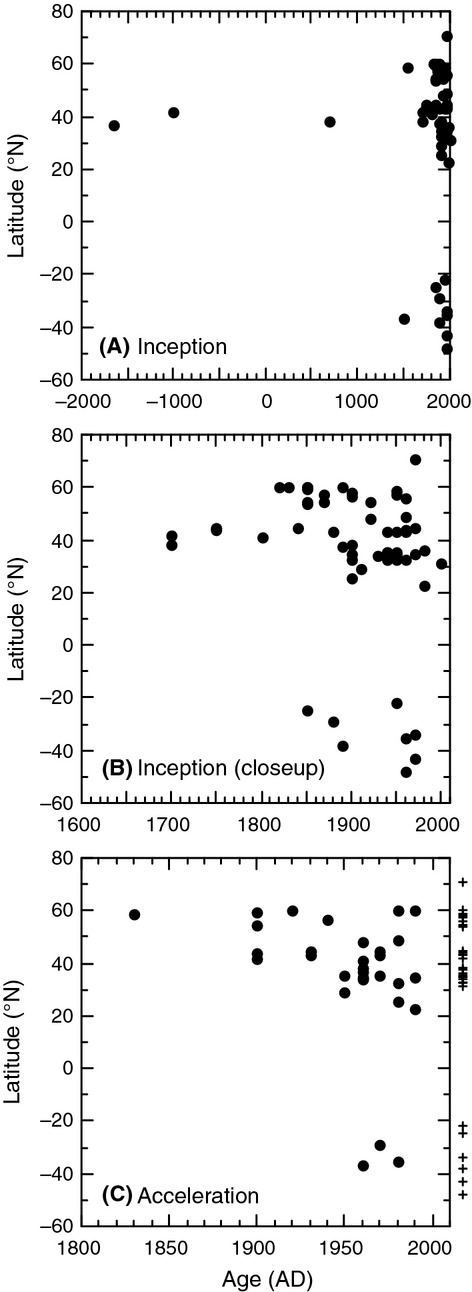
Latitudinal distribution of microfossil-based marine ecological degradation records. Latitude versus inception (A; B for closeup) and acceleration ages (C) of marine ecological degradation. Cross: locations lacking detectable acceleration. Data from Table S1.

**Figure 4:**
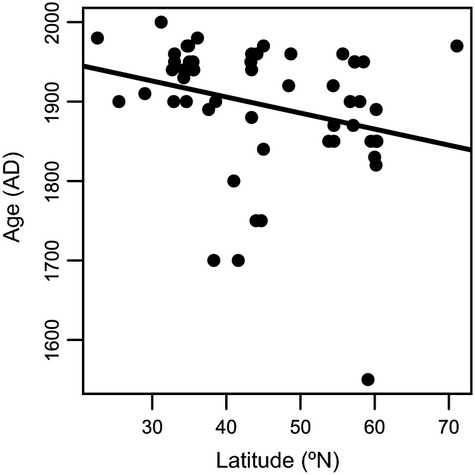
Relationship between latitude and the inception dates of marine ecological degradation in the northern hemisphere (*R*^2^ = 0.07362, *P* = 0.0494). Only inception dates >1000 AD were used.

**Figure 5:**
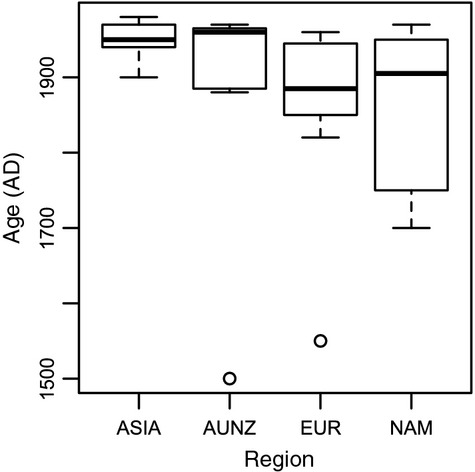
Boxplots showing regional differences of the inception dates of marine ecological degradation. ASIA: Asia; AUNZ: Australia and New Zealand; EUR: Europe; NAM: North America. Asian inception dates are significantly later than European (W = 261, *P* = 0.0008) and North American (W = 144, *P* = 0.0105) inception dates (but not significantly different from AUNZ inception dates: W = 36, *P* = 0.47). Only inception dates >1000 AD were used.

Most studies compiled here were conducted during or after the late 1990s (Fig. S1). A few pioneering studies go back to 1960–1970s, but their chronologies are often less reliable (Risdal 1963; Schafer 1970) because radiometric dating was not as widely available at that time, and thus are not included here. Microfossil records included in our analyses cover up to the last ∼10,000 years, but most of them cover only the last 200–300 years (Fig. 2). To evaluate regional differences in the timing of marine ecological degradation, we compared European, North American, Australia/New Zealand, and Asian records using Mann–Whitney tests. Datasets used for this paper are deposited at Dryad (http://dx.doi.org/10.5061/dryad.2t604).

## Global trends and regional differences of marine ecological degradation in microfossil records

### Distribution of microfossil records

In this review of microfossil records of ecological degradation, we focus on the timing of three events: the *inception* (i.e., start) of degradation, its *acceleration* (i.e., increasing severity of degradation), and when observed, *recovery* from a degraded state. Ideally, we would establish statistical means to define these events and apply them to the compiled case studies, but this approach is not feasible here because the raw data are not available for the vast majority of these studies. Accordingly, we take the more practical route of identifying these events through visual interpretation of published figures, with the identifications all performed by the lead author in order to maximize consistency. Care was taken in identifying inception of degradation as when biotic changes exceeded natural, background fluctuations. Ages of recovery were interpreted as the initiation of changes in community parameters returning toward prehistorical or pre-impacted states. Although this approach is necessarily somewhat subjective, it allows for comprehensive use of published information.

Evidence for marine ecological degradation in microfossil records was mostly found in shallow, coastal environments (Fig. 1; Table S1). Signs of ecological degradation were concentrated in North America, Europe, and East Asia (Fig. 1; Table S1) because strong human footprints, including hypoxic dead zones, are concentrated there (Diaz and Rosenberg 2008; Halpern et al. 2008), and because there are few studies in developing countries in Africa, Central and South America, and Southeast and West Asia. The temporal patterns of inception and acceleration of degradation and ecosystem recovery are illustrated in Figures 1–5 to characterize global trends.

### Inception of degradation

These data reveal that marine ecological degradation started in the 1800s in many regions of European and North American countries (Figs. 1–3), a pattern consistent with temporal patterns in industrialization and land clearing for agriculture in the 19th century, and with previous meta-analysis of fisheries, ecological, historical, archeological, and macrofossil data (Worm et al. 2006). The earliest degradation record extends back to ∼1500 BC (Figs. 1–3), however, and was caused by mining activity in the Tinto Estuary, Spain (Ruiz et al. 2009).

Northern hemisphere data clearly show an earlier start of marine ecological degradation in higher latitudes where developed countries are concentrated (Figs. 3–4). The start age of marine ecological degradation shows a noisy but significant relationship with latitude (*R*^2^ = 0.07362, *P* = 0.0494; Fig. 4). In addition, onsets of degradation occurred significantly later in Asia compared with Europe (W = 261, *P* = 0.0008) and North America (W = 144, *P* = 0.0105) (Fig. 5: only data for the past 1000 years are used for Mann–Whitney test). In the Black Sea, downcore evidence of introduced species by exploration or merchant ships extends back to two or three thousand years ago (Marret et al. 2009). In Europe, eutrophication footprints on faunal/floral composition extend back to ∼1550 in a Norwegian fjord (Alve 2000) and to the mid-19th century in the Baltic Sea (Andrén 1999; Andrén et al. 1999). Initial ecological degradation in the 1700s in Chesapeake Bay in North America is indicated by disturbances to diatom flora and submerged aquatic vegetation associated with European land clearance (Brush and Hilgartner 2000; Willard and Cronin 2007; Brush 2009). In contrast, no marine ecological degradation was detected from pre-1900 sediments in Asia. Instead, microfossil evidence indicated that degradation in Asia started rapidly and became widespread in the early to mid-20th century (Fig. 1), most likely related to Asian industrialization (as early as ∼1900, about 100 years later than European and North American industrialization) and post-World War II economic growth. Typical examples are found in Japanese embayments (Yasuhara et al. 2007; Katsuki et al. 2008; Tsujimoto et al. 2008). It is plausible that there were earlier instances of marine ecological degradation in Asia because large-scale land-use changes are known in Asia even back to the Neolithic period (Zong et al. 2007) and many Asian countries have long histories of well-developed cities. However, industrialization has had the dominant effect on sedimentary records reviewed here, although future work on core records that are both highly resolved and of extended duration will be needed to investigate if human-induced marine ecological degradation is detectable in pre-industrial Asia. Nevertheless, by the mid- to late-20th century, microfossil evidence of marine ecological degradation is globally widespread, occurring even in polar Greenland (Elberling et al. 2003) and a remote Pacific island (Debenay and Fernandez 2009) (Figs. 1 and 3).

### Acceleration of degradation

In the mid- to late-20th century, marine ecological degradation accelerated in many areas (Figs. 1–3) in concert with rapid post-World War II economic and population growth, and increased fertilizer use. The results show that the earlier acceleration occurred in higher latitudes, especially in the northern hemisphere (Fig. 3) where developed countries and cities are concentrated (see Overview of Regional Trends section).

### Ecosystem recovery

Alve (1995) presented a conceptual diagram that illustrates recovery from the degraded state following a similar (but reverse) trajectory as during the degradation process. However, the analysis of monitoring data in eutrophic systems by Duarte et al. (2009) indicates that degradation and recovery trajectories rarely coincide. Microfossil evidence indicates that relatively few sites show recovery from the degraded state to the predegradation state (e.g., abundance, diversity, and faunal or floral composition), and those recoveries that occurred were always during the late 20th century (Figs. 1 and 2) after various restoration efforts and environmental regulations were initiated. Furthermore, microfossil evidence indicates that complete recovery is very rare [see *European and African waters* section below for the Töölönlahti Bay study (Tikkanen et al. 1997) as one of the best examples of the ecosystem recovery]; instead post-recovery faunal or floral composition is usually different from predegradation faunal composition to varying degrees (McGann et al. 2003; Weckström 2006; Tsujimoto et al. 2008). Differences between degradation and recovery trajectories may occur because remediation of estuaries is usually slower than degradation (Hawkins et al. 2002), and because opportunistic species become the pioneer recolonizers under the post-degradation condition (Alve 1995). It is known that history of colonization (sequence of species arrival) influences community structure (Fukami and Morin 2003), and thus, “recovered” community is not necessarily same as predegradation community.

### Causes of marine ecological degradation

The predominant cause of degradation detected in microfossil records was nutrient enrichment and the resulting symptoms of eutrophication including hypoxia (Fig. S4). Other causes also played considerable roles in some areas, including severe metal pollution around mining sites, acidification by acidic wastewater, and salinity changes from construction of causeways, dikes, and channels, deforestation, and land clearance (Table S1). These environmental causes of marine ecological degradation are likely more prominent in microfossil records than reported in other recent reviews on ecological degradation (Jackson et al. 2001; Jackson 2008), because other reviews have mainly focused on relatively large animals in which fisheries exploitation overwhelms other causes.

## Nature of marine ecological degradation: paleoecological information from microfossils

Paleoecological signals (i.e., changes in microfossil assemblages) of marine ecological degradation can vary among systems, regions, and taxa (Table S1). There are, however, typical responses to particular stressors common among different locations in downcore microfossil records. Among these responses are changes in species diversity and total abundance, changes in abundance of both sensitive and tolerant species, increases in toxic algal bloom species, and the appearance or increase in invasive species.

### Species diversity

**Figure 6:**
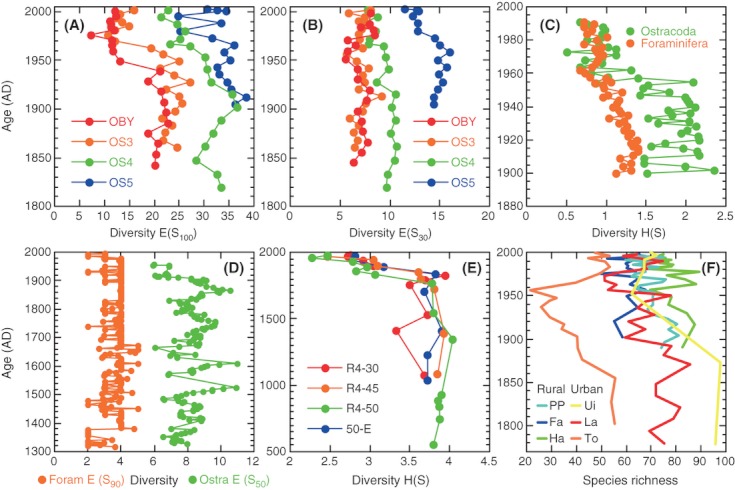
Long-term downcore trends of microfossil species diversity in representative regions from which high-resolution records are available. (A) Osaka Bay foraminiferal [data from Tsujimoto et al. (2008); OBY, OS3, OS4, and OS5: sediment core sites; cores OBY and OS3 were taken from inner part and cores OS4 and OS5 were taken from middle part of the bay], (B) Osaka Bay ostracod [data from Yasuhara et al. (2007)], (C) Gulf of Mexico foraminiferal (Blackwelder et al. 1996) and ostracod (Alvarez Zarikian et al. 2000), (D) Chesapeake Bay foraminiferal [data from Karlsen et al. (2000)] and ostracod [data from Cronin and Vann (2003)], (E) Chesapeake Bay diatom (Cooper 1995) (R4-30, R4-45, R4-50, and 50-E: sediment core sites), and (F) Baltic Sea diatom (Weckström et al. 2007) (PP, Fa, and Sa: rural sites; Ui, La, and To: urban sites) records. Species diversity shown by species richness, Shannon Index H(S), or the expected number of species in samples rarefied to *n* individuals E(S_n_) depending on availability.

**Figure 7:**
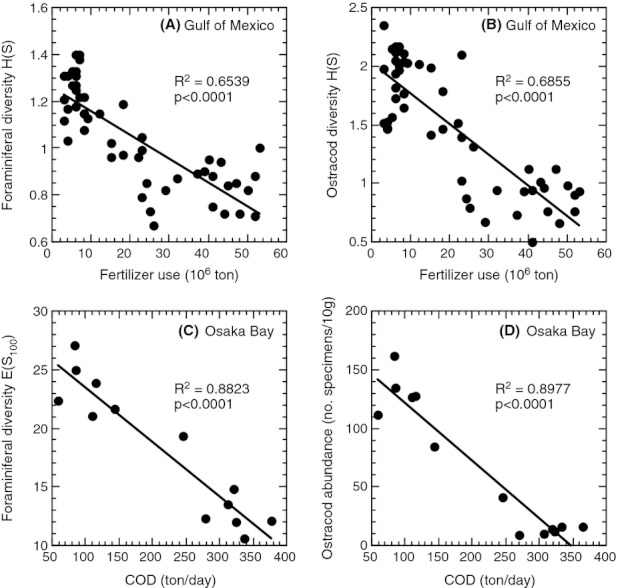
Relationship between eutrophication and species diversity and abundance. Correlations between (A) foraminiferal diversity H(S) in Gulf of Mexico core BC-10 and fertilizer use in USA, (B) ostracod diversity H(S) in Gulf of Mexico core BC-10 and fertilizer use in USA, (C) foraminiferal diversity E(S_100_) in Osaka Bay core OS3 and discharges of COD (chemical oxygen demand) from Osaka Prefecture, and (D) ostracod abundance (number of specimens per 10 g dry sediment) in Osaka Bay core OS3 and discharges of COD from Osaka Prefecture. Gulf of Mexico data from Alvarez Zarikian et al. (2000); Osaka Bay data from Yasuhara et al. (2007) and Tsujimoto et al. (2008).

A decline in species diversity is one of the typical signs of ecological degradation that can diminish ecosystem functioning (Cardinale et al. 2002; Worm et al. 2006; Danovaro et al. 2008). Species diversity is usually measured by the Shannon Index (H), species richness, or as the expected number of species in samples rarefied to *n* individuals E(S_n_). These measures of diversity decrease in most of the microfossil records from systems with strong human influence on water clarity, nutrient loads and oxygen concentrations. For example, foraminiferal diversity decreased in Osaka Bay, foraminiferal and ostracod diversity decreased in the Gulf of Mexico, and diatom diversity decreased in Chesapeake Bay (Fig. 6) after the onset of intense human alteration of the environment. These diversity decreases most likely result from increasing prey production and resulting dominance of a few opportunistic species, local extinctions due to decreasing bottom-water oxygen content, and reduced light penetration to the benthos (Rabalais et al. 2007; Tsujimoto et al. 2008; Brush 2009). In the Gulf of Mexico, both foraminifera and ostracod diversities showed significant, negative relationships with increasing US fertilizer use, and in Osaka Bay, foraminiferal diversity showed a significant, negative relationship with COD (chemical oxygen demand) discharge (Fig. 7). Although a direct oxygen paleo-proxy (i.e., geochemical evidence for low-oxygen content) is rarely available for comparison, these indirect evidences strongly support the above-mentioned hypothesis.

There are also some exceptions in which species diversity does not show a decreasing trend. In Osaka Bay, ostracod diversity is almost unchanged, especially in the inner part of the bay (i.e., cores OBY and OS3: Fig. 6), even though abundance and faunal composition show dramatic changes caused by eutrophication and hypoxia (Yasuhara and Yamazaki 2005; Yasuhara et al. 2007). This stability of ostracod diversity may reflect a low number of local extinctions, but the reasons behind the persistence of these species are uncertain. There is also no significant change in diversity observed in the foraminifera and ostracod records of the Chesapeake Bay (Fig. 6), although the low diversity throughout the core is likely related to this brackish environment that is naturally stressful for marine benthic organisms due to low and fluctuating salinity and other environmental factors.

Recovery of species diversity by various restoration efforts and environmental regulations is also observed in microfossil records, but only in a few locations (Ellegaard et al. 2006; Weckström et al. 2007; Alve et al. 2009; Fig. 6). Instead, in most locations, diversities in the most recent sections of cores were significantly lower than those in pre-industrial or pre-urbanization core sections: For example, Mann–Whitney test shows that foraminiferal diversity is significantly (*P* < 0.05) higher in pre-urbanization (<1950) samples than in post-urbanization (>1950) samples in Osaka Bay cores (Fig. 6A), except core OS5 (*P* = 0.073) that has much smaller sample size and locality far from the source of anthropogenic nutrient input (the mouth of the Yodo River) compared with other cores.

### Abundance

**Figure 8:**
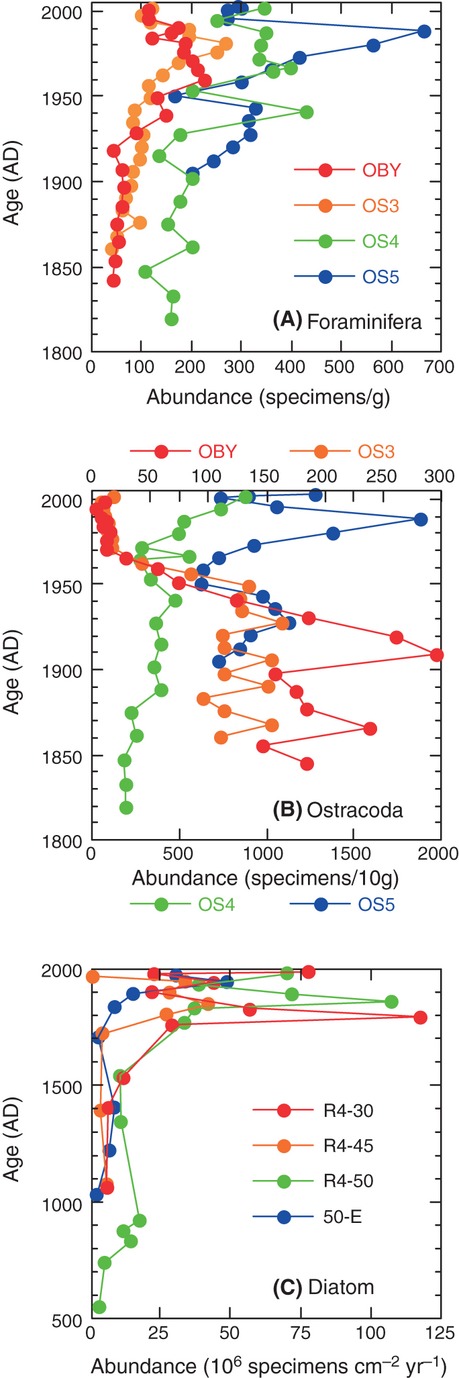
Long-term downcore trends of microfossil abundance in representative regions from which high-resolution records are available. (A) Osaka Bay foraminiferal (Tsujimoto et al. 2008) and (B) Osaka Bay ostracod (Yasuhara et al. 2007) records (OBY, OS3, OS4 and OS5: sediment core sites; cores OBY and OS3 were taken from inner part and cores OS4 and OS5 were taken from middle part of the bay) and (C) Chesapeake Bay diatom records (Cooper 1995) (R4-30, R4-45, R4-50, and 50-E: sediment core sites). In Osaka Bay, ostracode abundance decreases in the inner part of the bay but increases in the central part of the bay, because Osaka Bay hypoxia is restricted in the inner part of the bay (see Overview of Regional Trends section for mechanism of abundance change).

Marine ecological degradation is also detected as a decrease or increase in abundance, depending on the taxonomic group, habitat, and physiological tolerances of individual species (Fig. 8). In coastal ecosystems, anthropogenic causes of abundance changes in microfossil assemblages are mainly eutrophication and the resulting bottom-water hypoxia and/or anoxia (e.g., Cooper 1995; Yasuhara et al. 2007; Tsujimoto et al. 2008; Table S1). Plankton usually increases in response to an increasing nutrient and prey supply associated with eutrophication, a few benthic species that are resistant to low-oxygen conditions and low light penetration also increase for the same reason, and most benthic species that cannot tolerate low-oxygen conditions increase in the case of eutrophication without hypoxia or anoxia, but decrease where hypoxia or anoxia result from eutrophication. For example, total diatom and dinoflagellate abundances increase because of a substantial increase in planktic species; foraminiferal abundance increases because of a substantial increase in a few resistant species to hypoxic conditions; and ostracod abundance increases or decreases depending on oxygen concentrations (no ostracod species thrive in hypoxic conditions; the relative abundance of a few relatively resistant species increases but their absolute abundance still decreases). Absolute abundance information was rarely reported in the microfossil records that we compiled here, but the above-mentioned trends seem to be typical (Fig. 8; Table S1). Although quantitative proxies of eutrophication and/or oxygen content are not usually available for direct comparison with microfossil paleoecological data, a significant negative relationship was found between ostracod abundance in a hypoxic site of Osaka Bay and COD discharge from the Osaka Prefecture through the Yodo River to the bay (Fig. 7).

### Taxon-specific responses

#### Benthic foraminifera

**Figure 9:**
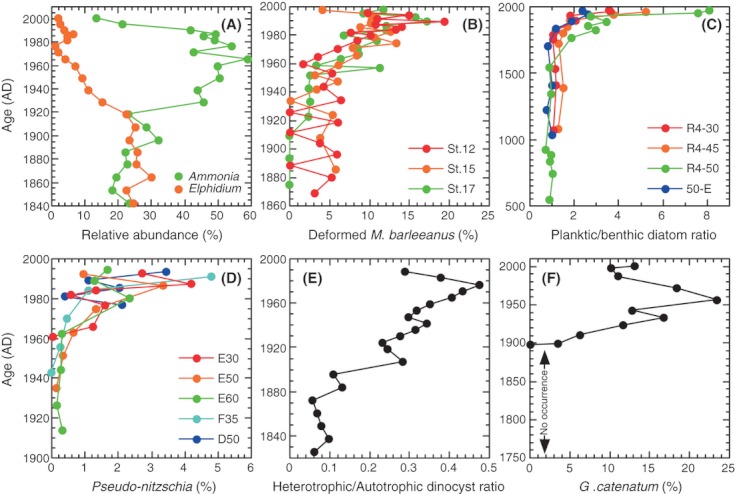
Representative downcore faunal and floral changes. Relative abundance of foraminiferal genera, *Ammonia* and *Elphidium*, in core OBY, Osaka Bay (Tsujimoto et al. 2008) (A); relative abundance of deformed specimens of foraminiferal species, *Melonis barleeanus* in sediment cores St. 12, St. 15, and St. 17 in a Greenlandic fjord (Elberling et al. 2003) (B); Planktic/benthic diatom ratio in Chesapeake Bay cores R4-30, R4-45, R4-50, and 50-E (Cooper 1995) (C); relative abundance of toxic diatom genus, *Pseudo-nitzschia*, in Gulf of Mexico cores E30, E50, E60, F35, and D50 (Parsons and Dortch 2002) (D); Heterotrophic/Autotrophic ratio of dinoflagellate cysts in the Adriatic Sea (Sangiorgi and Donders 2004) (E); and relative abundance of introduced dinoflagellate species, *Gymnodinium catenatum*, in Portuguese Margin, North Atlantic (Amorim and Dale 2006) (F).

In benthic foraminifera, the tolerant genus *Ammonia* (here meaning tolerant of hypoxia/anoxia or preferring eutrophication and food-rich environment) increases and the sensitive genus *Elphidium* decreases with increasing eutrophication and resulting hypoxia/anoxia at various locations, including Osaka Bay (Tsujimoto et al. 2006, 2008), Gulf of Mexico (Sen Gupta et al. 1996; Sen Gupta and Platon 2006; Rabalais et al. 2007), Chesapeake Bay (Karlsen et al. 2000), San Francisco Bay (McGann 2008), Long Island Sound (Thomas et al. 2000), and the Bay of Biscay (Irabien et al. 2008). For example, in Osaka Bay, *Ammonia* starts to increase and *Elphidium* starts to decrease immediately after the industrial revolution in Japan at ∼1900 (Fig. 9). Agglutinated foraminiferal taxa are also known to be tolerant and increase in various regions (Alve 1996; Hayward et al. 2004; Tsujimoto et al. 2008). The tolerant species *Eggerella advena* and *Trochammina hadai* are also widely distributed and dominant in degraded ecosystems (McGann et al. 2003; Katsuki et al. 2008; Tsujimoto et al. 2008). Note that it is difficult to disentable the separate effects of oxygen depletion from other symptoms of eutrophication because we lack finely resolved environmental proxies for each.

#### Benthic Ostracoda

There is no universal response by particular benthic ostracod taxa to ecological degradation, probably in part because the limited dispersal abilities of these animals result in regionally differentiated faunas. There are, however, several tolerant species known in each region, for example, *Loxoconcha* sp. in the eastern coast of USA (Alvarez Zarikian et al. 2000; Cronin and Vann 2003) and *Bicornucythere bisanensis* in Japan (Irizuki et al. 2003; Yasuhara et al. 2003, 2007).

#### Diatoms and dinoflagellates

Eutrophic ecosystems are also characterized by the dominance of planktic diatoms and the decline of benthic diatoms (Clarke et al. 2006; Katsuki et al. 2009; Tuovinen et al. 2010), and by the increased dominance of heterotrophic dinoflagellates at the expense of autotrophic species (Dale 2000; Matsuoka 2001; Sangiorgi and Donders 2004), although a few exceptions are known (Chmura et al. 2004; Liu et al. 2008). In Chesapeake Bay, the planktic/benthic diatom ratio increased dramatically after European settlement and land clearance at ∼1700 (Cooper 1995) (Fig. 9). In the Adriatic Sea, the heterotrophic/autotrophic dinoflagellate cyst ratio increased beginning in ∼1900 due to a progressive increase in eutrophication (Sangiorgi and Donders 2004) (Fig. 9). An increase in *Chaetoceros* diatom cysts is also a typical signal of eutrophic ecosystems (Miller and Risberg 1990; Andrén 1999; Andrén et al. 1999).

#### Toxic bloom, introduced species, and deformity

**Figure 10:**
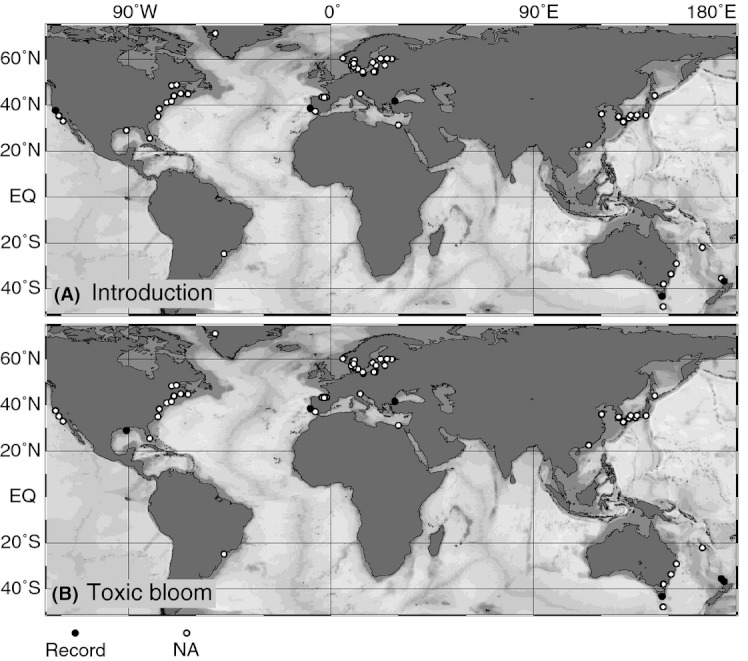
Global distribution of marine ecological degradation in downcore microfossil records. Distribution of (A) introduction and (B) toxic bloom events. NA: site with no introduction or toxic bloom evidence. Data from Table S1.

Recent increases in toxic blooms and introduced species are recorded in microfossil records from several sites in the USA (McGann and Sloan 1996; McGann et al. 2000; Parsons and Dortch 2002), Europe (Amorim and Dale 2006; Marret et al. 2009), Australia (McMinn et al. 1997), and New Zealand (Irwin et al. 2003; Grenfell et al. 2007) (Fig. 10). For example, in the Gulf of Mexico, the toxic diatom genus *Pseudo-nitzschia* has rapidly increased since 1960 (Parsons and Dortch 2002) (Fig. 9) and the toxic dinoflagellate *Gymnodinium catenatum* has been introduced into the eastern Atlantic at ∼1900 via ballast water (Amorim and Dale 2006) (Fig. 9). Late-20th century introductions and blooms of this species are also known in Australia and New Zealand (McMinn et al. 1997; Irwin et al. 2003). On the western coast of the USA, introduction of the Japanese foraminiferal species *Trochammina hadai* is also known from ∼100 years ago (McGann 2008) and other benthic foraminiferal introductions are known in New Zealand (Grenfell et al. 2007). The earliest record of introduced microfossil species is from the southwestern Black Sea shelf at ∼1000 BC, possibly associated with early Greek exploration (Marret et al. 2009).

Deformed specimens of foraminifera also increase with increased human-induced stresses such as pollution by heavy metals (Elberling et al. 2003) and polychlorinated biphenyls (Scott et al. 2005) (Fig. 9) and hypoxia/anoxia (Karlsen et al. 2000), although the ultimate cause of foraminiferal deformity is still controversial (Polovodova and Schönfeld 2008; Martinez-Colon et al. 2009). In Aburatsubo Cove, Japan, specimens of ostracod *Bicornucythere bisanensis* with variant carapace ridges are relatively abundant (∼10–20%), which may be related to pollution because such variant ridges are never found in Holocene and Pliocene fossil specimens (Abe 1983).

## Overview of regional trends

### North American waters

In North America, the marine ecological degradation records are concentrated in the Atlantic Coast (e.g., Chesapeake Bay, Long Island Sound, and Canadian harbors), and Gulf of Mexico. In Chesapeake Bay, initial signs of ecological degradation are recognizable as an increase in total diatom abundance and in the diatom planktic/benthic ratio at around 1700, as a consequence of European settlement and deforestation and resulting increased river discharge and initial eutrophication (Cooper 1995; Cronin and Vann 2003). Later at ∼1800, diatom diversity started to decrease (Cooper 1995), because of further deforestation (Cronin and Vann 2003; Willard and Cronin 2007; Brush 2009) and perhaps increased human activity related to industrialization. However, the most drastic changes occurred ∼1960 due to urbanization and substantially increased population and fertilizer use (Cronin and Vann 2003; Willard and Cronin 2007; Brush 2009). The resulting eutrophication, hypoxia, and anoxia have become much more severe and widespread since this time, with an accelerated increase in the diatom planktic/benthic ratio and changes in the ostracod and foraminiferal fauna (Cooper 1995; Karlsen et al. 2000; Cronin and Vann 2003; Willard and Cronin 2007; Brush 2009).

A core drilled in the bottom of the deep channel provided a continuous high-resolution paleoecological record for the last 8000 years (Fig. S5) and clearly showed the unique nature of the mid–late 20th century ecosystem. The ostracod *Loxoconcha* sp. is solely dominant throughout most of the last 8000 years. Since this species is known to be common in hypoxic environments (Alvarez Zarikian et al. 2000),), the continuous dominance of this species suggests that the Chesapeake Bay deep benthic ecosystem has experienced hypoxia to some degree for at least the last 8000 years even without human influence. Currently, anoxia and hypoxia are widespread in the Chesapeake Bay. *Cytheromorpha curta,* another species that is known to be hypoxia-tolerant or opportunistic (Cronin and Vann 2003) and was never found in pre-1940 sediments, rapidly became dominant beginning in the mid-20th century, although *Loxoconcha* sp. is also still common (Fig. S5). This faunal change likely represents further ecological degradation. Ostracod and foraminiferal data in a shallower, channel edge core, which were taken in an area that currently experiences hypoxia, show a similar trend, in which *Cytheromorpha curta* and the foraminiferal species *Ammonia parkinsoniana*, rare or absent in pre-20th century sediments, became dominant in the mid–late 20th century (Karlsen et al. Cronin and Vann 2003). The relative abundance of deformed shells of *Ammonia parkinsoniana* also increased in this period. The relative abundance of *Loxoconcha* sp. showed multi-decadal–centennial oscillations ranging from 0 to 40% in pre-20th century sediments (Cronin and Vann 2003), suggesting that the shallower benthic ecosystem was only periodically hypoxic, in contrast with the deep channel [Similar patterns are also known in the Gulf of Mexico (Platon et al. 2005; Osterman et al. 2009)]. Currently, *Loxoconcha* sp. is widely dominant in the deep channel of the Chesapeake Bay (Cronin and Vann 2003).

Microfossils also provide evidence of post-1960s ecological degradation in the Gulf of Mexico (Blackwelder et al. 1996; Sen Gupta et al. 1996; Alvarez Zarikian et al. 2000; Osterman et al. 2005; Platon et al. 2005; Rabalais et al. 2007; Osterman et al. 2008, 2009). Ostracod and foraminiferal diversity rapidly decreased beginning in ∼1960 (Blackwelder et al. 1996; Alvarez Zarikian et al. 2000; Rabalais et al. 2007). At the same time, tolerant or opportunistic foraminiferal (e.g., *Ammonia parkinsoniana*), ostracod (e.g., *Loxoconcha* sp.), and diatom (e.g., *Pseudo-nitzschia pseudodelicatissima,*) species became dominant and other (sensitive) species (e.g., *Elphidium* spp. for foraminifera) collapsed (Sen Gupta et al. 1996; Alvarez Zarikian et al. 2000; Parsons and Dortch 2002; Osterman et al. 2005; Platon et al. 2005; Rabalais et al. 2007; Osterman et al. 2008, 2009). These faunal changes are associated with eutrophication and hypoxia resulting from population growth, urbanization, and increased use of fertilizers in the post-World War II era (Rabalais et al. 2002, 2007). Similar post-World War II ecological degradation is known from the Neuse and Pamlico River estuaries (Cooper et al. 2004), Long Island Sound (Thomas et al. 2004), Canadian harbors, estuaries, and fjords (Schafer et al. 1991; Scott et al. 1995, 2005; Thibodeau et al. 2006), and the western coast of North America of Southern California Bight (McGann et al. 2003; McGann 2009) and San Francisco Bay (McGann 2008).

Ecosystem impacts of land clearance and deforestation is also found in other microfossil records in North American waters, for example, in New Bedford Harbor as an increase in autotrophic dinoflagellates (Chmura et al. 2004), in Merrymeeting Bay as an increase in diatom abundance (Köster et al. 2007), and in Halifax Harbour as an increase in foraminiferal and dinoflagellate abundance (Miller et al. 1982). Microfossil assemblages, such as foraminifera, diatom, and dinoflagellate cyst, also provide evidence of urbanization impacts appeared as early as ∼1900 in shallow-marine ecosystems adjacent to several cities, including New Bedford Harbor (Pospelova et al. 2002; Chmura et al. 2004; Scott et al. 2005) and Merrymeeting Bay (Köster et al. 2007).

### European and African waters

In Europe, numerous studies have been conducted in the Baltic Sea, mainly using diatoms. In the urban sites of the Gulf of Finland, increases in the relative abundance of planktic diatoms reflect urbanization-induced eutrophication beginning early in 19th century (Weckstrom et al. 2004; Kauppila et al. 2005; Weckström 2006). After this initial degradation, species diversity started to decrease starting in the late 19th century, and planktic and eutrophication-indicator species are dominant throughout the 20th century (Weckstrom et al. 2004; Kauppila et al. 2005; Clarke et al. 2006; Weckström 2006; Weckström et al. 2007). A clear recovery of species diversity is observed since the 1970s related to the cessation of wastewater loading, although planktic diatoms are still dominant (Kauppila et al. 2005; Clarke et al. 2006; Weckström 2006; Weckström et al. 2007). In contrast, the ecological degradation observed in the rural sites of the gulf was minor, and is reflected in the microfossil record as an increasing relative abundance of planktic diatoms and a slight diatom diversity decrease (but not always clear); these changes occurred later, starting in the 1940s and accelerating in 1980s, mainly due to intensification of agriculture in Finland after the World War II (Weckström 2006; Weckström et al. 2007). Similar trends are found in other locations in the Baltic Sea (Miller and Risberg 1990; Grönlund 1993; Witkowski and Pampkowiak 1995; Korhola and Blom 1996; Tikkanen et al. 1997; Andrén 1999; Andrén et al. 1999, 2000; Jankowska et al. 2005; Witak et al. 2005; Witak and Jankowska 2005; Witak et al. 2006; Leśniewska and Witak 2008; Olli et al. 2008; Tuovinen et al. 2010). Töölönlahti Bay is one of the best examples of marine ecosystem recovery observed in the microfossil record (Tikkanen et al. 1997). Species diversity and faunal composition almost completely recovered to the 19th century condition after cessation of waste-water disposal in the 1960s (Tikkanen et al. 1997).

Intensive research has also been conducted in northern European fjords mainly using foraminifera and dinoflagellate cysts. Available evidence suggests that marine ecological degradation extends back to mid 16th century in this region. In Frierfjord, Norway, marine ecological degradation probably started at ∼1550 associated with initial eutrophication caused by saw mills (Alve 2000; Dale 2000). In Oslofjord, Norway, initial ecological degradation dates to 1850, as evidenced by a decrease in foraminiferal species diversity and an increase in the abundance of total dinoflagellates and *Lingulodinium machaerophorum;* this was caused by eutrophication associated with human population growth (Nagy and Alve 1987; Alve 1991; Dale and Fjellså 1994; Dale et al. 1999; Dale 2000; Alve et al. 2009). After that, from the early to mid 20th century, a rapid increase in the abundance of total foraminifera occurred, together with an increase in tolerant, opportunistic, and arenaceous foraminiferal species and a decrease in sensitive and calcareous foraminiferal species (Nagy and Alve 1987; Alve 1991; Alve et al. 2009). Ecosystem recovery began in the 1970s as a result of relocating much of the Oslo City's sewage treatment (Dale et al. 1999; Alve et al. 2009). Similar ecological degradation histories are known from microfossil records in other North European fjords (Moodley et al. 1993; Paetzel and Schrader 1995; Alve 1996; Hass 1997a,b; Thorsen and Dale 1997; Amsinck et al. 2003; Clarke et al. 2003; Ryves et al. 2004; Clarke et al. 2006; Ellegaard et al. 2006). In Limfjord and Vejlerne Nature Reserve, Denmark, remarkable faunal and floral changes were caused by salinity decrease due to land reclamation and closure of the fjord in the 1870s (Amsinck et al. 2003; Ryves et al. 2004).

In Europe, microfossils also record marine ecological degradation in Spanish estuaries caused by acidic wastewater, metal pollution, eutrophication, oxygen depletion, and increased sedimentation as a result of mining and smelting operations, chemical factories, petroleum refineries, industrialization, and land reclamation (González-Regalado et al. 1996; Ruiz Muñoz et al. 1997; Cearreta et al. 2000; Gonzalez-Regalado et al. 2001; Cearreta et al. 2002a,b; Pascual et al. 2002; Ruiz et al. 2004; Irabien et al. 2008; Ruiz et al. 2008, 2009). In Portugal, ecosystem changes in the Albufeira Lagoon were caused by salinity increase as a result of anthropogenic alterations of the barrier (Bao et al. 1999); whereas on the Atlantic Portuguese coast, changes were caused by urbanization-induced eutrophication and pollution (Amorim and Dale 2006; Bartels-Jónsdóttir et al. 2006, 2009). In the Adriatic Sea, changes were caused by eutrophication and anoxia as a result of human activities including agriculture, wastewater disposal, and diversion of river outflow (Puskaric et al. 1990; Barmawidjaja et al. 1995; Sangiorgi and Donders 2004).

There are no African data available, except for foraminiferal research along the Mediterranean coast of Egypt, where large faunal change was caused by causeway reconstruction ∼2000 years ago (Bernasconi et al. 2006).

### Asian waters

In Asia, most late Holocene marine micropaleontological records are from Japan. For example, in Osaka Bay, microfossils indicate that marine ecological degradation started with the beginning of the Japanese industrialization period at ∼1900. Foraminiferal diversity and abundance started to decrease and increase, respectively, and a decline of sensitive species and increase in tolerant or opportunistic species also started at that time. At the same time ostracod faunal patterns changed in a similar fashion, and ostracod abundance decreased in the inner part the bay. During the Japanese high economic growth period of 1950s–1970s, these foraminiferal and ostracod faunal trends accelerated. The degradation of Osaka Bay was mainly caused by urbanization-induced eutrophication and resulting bottom-water hypoxia. In the middle part of the bay, ostracod abundance increased, especially during the mid–late 20th century, due to increased nutrient supply without hypoxia. In the inner part of Osaka Bay, the abundance of the opportunistic foraminiferal species decreased and the foraminiferal species diversity increased since the 1970s. This recovery was a result of environmental regulation, which led to a reduction in organic pollution loads and a decrease in food supply to the benthos. However, modern foraminiferal distribution is still dominated by the opportunistic species. In addition to foraminiferal and ostracod records, dinoflagellate microfossils are also abundant in Japanese and Korean embayments, and show eutrophication-induced increases in heterotrophic species during the early to mid 20th century (Matsuoka 1995; Kim and Matsuoka 1998; Matsuoka 1999; Matsuoka and Kim 1999; Matsuoka 2001; Kim et al. 2009; Matsuoka and Shin 2010; Shin et al. 2010).

There are also many data available from Japanese brackish lakes. In Lake Nakaumi, significant ecological degradation had occurred since the 1940s due to enhanced salinity variability and bottom-water hypoxia as a result of construction projects including land reclamation and dike construction, as well as human-induced eutrophication. Benthic diatom disappeared after the 1940s, red-tide diatom became dominant in 1950s -1960s, and foraminiferal and ostracod faunas had altered since 1940. In Lake Saroma, scallop farming since the 1960s has caused intensive eutrophication and bottom-water hypoxia and resulting ecological degradation, that is represented by increase and decrease in the planktic and benthic diatoms, respectively.

Elsewhere, microfossils recording 20th century eutrophication-induced marine ecological degradation have also been analyzed in Ariake and Isahaya Bays, Japan (Akimoto et al. 2004; Matsuoka 2004; Yokose et al. 2005); in Hiroshima Bay, Japan (Yasuhara et al. 2003); in Tokyo Bay, Japan (Matsumoto 1981, 1983; Matsumoto and Saito 1984; Ikeya 1995; Sato 1995; Toyoda and Kitazato 1995; Matsuoka 1999; Tanimura et al. 2003); Lake Kugushi, Japan (Nomura and Kawano 2011); Jiaozhou Bay, China (Liu et al. 2008); and Daya Bay, South China Sea (Wang et al. 2004).

### Southern hemisphere waters

In the southern hemisphere, the few late Holocene marine microfossil studies conducted are concentrated in New Zealand and Australia. In Australia, salinity-change-induced ecological degradation and introduction of exotic species are recorded for the eastern coast, which were caused by construction and ballast water release, respectively, during the late 19th–20th century (McMinn et al. 1997; Saunders et al. 2007, 2008; Taffs et al. 2008; Saunders and Taffs 2009). New Zealand studies in the North Island harbors indicated that Polynesian forest clearance caused minor but initial ecological degradation starting at ∼1500 (Hayward et al. 2006). European land clearance since ∼1840 and late-20th-century urbanization had a much larger impact on the marine ecosystem (Hayward et al. 2004; Matthews et al. 2005; Hayward et al. 2006; Grenfell et al. 2007; Hayward et al. 2008). Details of North Island harbors' ecological degradation is complex and involves industrial and domestic sewage disposal, introduction of Asian date mussels and cordgrass, land clearance/deforestation, urbanization, and oyster farming (see, Hayward et al. 2004; Matthews et al. 2005; Hayward et al. 2006; Grenfell et al. 2007; Hayward et al. 2008).

In South America, the only available data are from the Atlantic coast of Brazil (de Mahiques et al. 2009). Foraminiferal faunal changes including increase in agglutinated species and decrease in calcareous species, species diversity, and abundance in that region were likely caused by salinity decrease and heavy metal pollution as a result of opening of an artificial channel and mining activities since the 1850s (de Mahiques et al. 2009).

## Problems, solutions, and outlook of marine paleoecological approach based on microfossils

As reviewed in the present paper, microfossil paleoecology is useful for marine ecological degradation research. However, there are various limitations and concerns inherent to this approach. In this section, we discuss these issues and their possible solutions, along with the outlook for developing microfossil-based marine ecological degradation research in the future.

### Climate versus human-induced environmental changes

Although our micropaleontological literature review strongly suggests that most instances of marine ecological degradation are caused by eutrophication and resulting hypoxia, many of the studies are qualitative and their interpretation is subject to some uncertainties. Furthermore, these studies generally do not take into account possible effects of climatic changes, including major events such as the Little Ice Age and 20th century global warming, although a few exceptions use statistical approaches to do so (e.g., Uthicke et al. 2012). Although changes in high-human footprint habitats such as urban embayments are not easily attributable to climate cooling and warming, the possibility that ecological degradation is related to climatic change merits further consideration, focusing on these two climatic events.

#### Little Ice Age

The Little Ice Age (AD ∼1400–1700) is the most recent major climatic cooling event (Mann et al. 2009). In the Baltic Sea, Andrén et al. (2000) reported a detectable influence of the Little Ice Age on fossil diatom assemblages. However, clear indications of the Little Ice Age (e.g., increase in cold water indicator species) are rarely detected in microfossil faunal and floral records. In fact, no other research in the Baltic Sea has detected a clear paleoecological footprint of the Little Ice Age. In the Chesapeake Bay, trace element analysis of ostracod shells clearly showed Little Ice Age cooling (Cronin et al. 2005; Willard and Cronin 2007; Cronin et al. 2010). Although the climate change was clearly present in geochemical records, no clear biotic response could be detected in marine microfossil communities (Karlsen et al. 2000; Cronin and Vann 2003). Furthermore, the magnitude of temperature decline during the Little Ice Age is relatively small (<1°C) (Mann et al. 2009) and thus it is unlikely that recent microfossil evidence of ecological degradation reviewed in the present paper is mainly caused by this cooling event.

#### Global warming

Recent research suggests that human-induced global warming negatively affects marine biodiversity (Harley 2011). However, ecological theories suggest positive relationship between temperature and biodiversity both on evolutionary and ecological time-scales (Allen et al. 2002; Currie et al. 2004; Yasuhara et al. 2012b). Thus, it is still largely uncertain how global warming affects marine ecosystems.

Furthermore, global warming since ∼1900 has increased temperatures by less than 1°C (Intergovernmental Panel on Climate Change 2007), which is small compared with seasonal and yearly fluctuations [although some habitats, such as coral reefs, may be particularly sensitive to warming (e.g., Pandolfi et al. 2011)]. Moreover, many of the ecosystem degradation events compiled here predate 20th century warming. In addition, the autoecology of affected taxa, and extrinsic information about land use and environmental change consistently suggest that eutrophication and the resulting hypoxia is the predominant cause of marine ecosystem degradation as reviewed in this article. Recent studies suggest that global-warming footprints on marine ecosystems are detectable from the systems much less influenced by human-induced nutrient enrichment, for example, pelagic, open ocean (Field et al. 2006; Spielhagen et al. 2011). Nevertheless, much of this evidence is qualitative in nature, and we discuss below how to improve this situation. Climatic change may also be related to eutrophication itself (Savage et al. 2010), and the connection between these two phenomena warrants further research.

### Temporal resolution, time-averaging, and taphonomic bias

Robust chronology is essential for the accurate reconstruction of the past ecological changes from sedimentary records. There are various tools for inferring age-depth relationships within cores spanning hundreds to thousands of years. Radiometric dating methods, for example ^210^Pb, ^137^Cs, and ^14^C, are widely used on this time scale. Radiocarbon (^14^C) dating has ∼10^1^–10^2^ years error and thus does not have sufficient resolution for the past 100–200 years sediments, but it is useful for older sediments. In contrast, ^210^Pb and ^137^Cs methods are applicable only for the past ∼100–200 years sediments, because half-life of ^210^Pb is short and ^137^Cs method is based on detection of nuclear testing or accident events. Other dating methods including pollen biostratigraphy and tephrochronology are also often useful, depending on the regions and time intervals. Thus, usage of multiple complementary methods is important for reconstructing robust chronology throughout the past 10^2^–10^3^ years (e.g., Willard et al. 2003). Furthermore, selecting high sedimentation rate sites are important to obtain high time-resolution records. For further details of dating methods, see Cronin (2010).

Bioturbation and physical mixing combines fossils from different intervals of time and thereby places a practical limit on the temporal resolution of sediment records. Under most conditions, yearly resolution is not attainable, but decadal-scale resolution can be, as long as the sedimentation rate is high enough (e.g., Cronin et al. 2010). Fortuitously, bottom-water hypoxia tends to increase the temporal acuity of sedimentary records because few bioturbating animals can tolerate very low-oxygen levels. Thus, cores recording marine ecological degradation from this cause tend to record decadal signal well, especially during degraded periods.

Microfossil shells are small and thus can be transported by current after their death. Such postmortem transportation is more likely at sites dominated by coarser (i.e., sandy) sediments that indicate deposition under stronger currents than at muddy sites typical of low-energy environments. Although rarely applied, there are several ways proposed to detect such postmortem transport (e.g., see Whatley 1988 for ostracods).

Selective preservation is also a potential problem, especially in higher energy environments. Microfossil species with robust shells will be better preserved than those with thin, delicate shells. Shells of earlier juveniles tend to be thinner and more delicate, that is also a potential cause of selective preservation. However, microfossils are still generally very well preserved in sedimentary records compared with any other fossil groups. Preservation states of microfossils can be tested by, for example, fragmentation rate and clarity (Martin 1999; Dwyer et al. 2002; Sexton et al. 2006).

Time-averaging, selective preservation, and transport may render fossil assemblages biased samples of their source live communities, but intensive assessments show good fidelity between living organisms and the death assemblage of shells for mollusks (Kidwell 2001; Tomašových and Kidwell 2009). Such live-death fidelity is also known in microfossils (e.g., Kornicker 1964; Izuka and Kaesler 1986; Wollenburg and Kuhnt 2000). Time-averaging can even be an advantage in ecological research. Fossil samples typically represent more than 1 year (usually a few to tens of years, depending on sedimentation rate and sample thickness). This multi-year averaging means that variability at shorter time-scales (i.e., hourly, daily, and seasonal) can be eliminated (Olszewski 1999). This temporal smoothing is an advantage because, in the context of ecological degradation, such short-term variation is usually just noise around what is of much greater interest – shifts in the long-term state of the community. Substantial effort can be required to determine annual or multi-year average conditions when sampling modern biota (see Kidwell 2009).

### Interpretation of paleoecological records: beyond narrative interpretation and toward more quantitative and statistical approach

In our review of these microfossil records, our interpretations about the inception and acceleration of degradation were necessarily qualitative because the raw data are not available for most studies, and because many of the original studies were essentially descriptive. However, several studies used multivariate analyses and visualization methods effectively.

**Figure 11:**
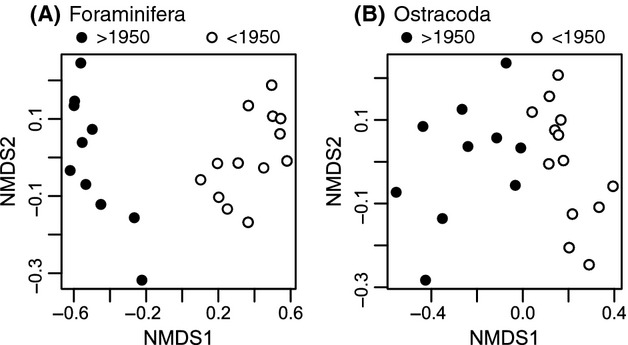
Ordination (NMDS) of microfossil relative abundance data in Osaka Bay core OS3. (A) foraminifera. (B) ostracods. Solid circles: post-1950 samples. Open circles: pre-1950 samples. Data from Tsujimoto et al. (2008) and Yasuhara et al. (2007).

Changes in faunal and floral composition are typical of marine ecological degradation. Such signs can be detected as changes in specific species or genera as reviewed in the *Taxon-specific responses* section. But, multivariate analyses such as cluster analysis, nonmetric multidimensional scaling (NMDS), and detrended correspondence analysis (DCA) are important for summarizing and detecting whole-assemblage composition changes (Murray 2000; Yasuhara et al. 2003; Tuovinen et al. 2010; Uthicke et al. 2012). For example, in the NMDS plots of Osaka Bay core OS3, pre-1950 and post-1950 samples segregate neatly, showing the transition from pre-urbanization to post-urbanization assemblages coincident with Japan's high economic growth and resulting eutrophication and hypoxia after World War II (Fig. 11).

**Figure 12:**
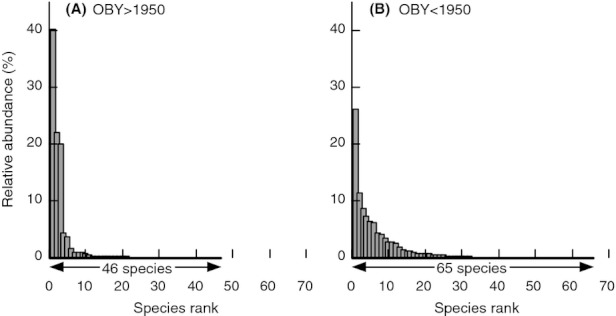
Rank-abundance distributions of foraminifera in Osaka Bay core OBY. (A) post-1950 assemblage (46 species in total). (B) pre-1950 assemblage (65 species in total). Data from Tsujimoto et al. (2008).

Rank-abundance distributions can also show clear evidence of altered faunal composition (Scott et al. 2001). In Osaka Bay core OBY, rank-abundance distributions were remarkably different between pre- and post-urbanization (∼1950) faunas (Fig. 12). Pre-urbanization faunal composition has a relatively equitable distribution of abundances from dominant to rare species, with a long tail of rare species. In contrast, the post-urbanization fauna is dominated by only three resistant species at the expense of the rest of the fauna, with lower species richness overall. Although more richly informative than descriptive approaches, these more quantitative methods have been applied in only a minority of published studies.

Ecosystem history research and microfossil-based reconstruction of environmental history may sometimes be two sides of the same coin. For example, we can interpret biodiversity decline and other biotic changes as ecological degradation, but the same information can be used as an indicator of pollution. This equivalence assumes a direct, immediate, and linear response between environmental changes and biotic response of microfossil communities. In the absence of independent proxies for environmental history, these assumptions cannot be tested. It is therefore an important priority for future work to document changes in assemblages jointly with geochemical and other independent proxies for environmental alteration (see e.g., Brush 2008). Moreover, it will be most productive to do so in a rigorous statistical framework, which is one aspect in which marine paleoecological research lags behind paleolimnology (e.g., Cottingham et al. 2000; Rusak et al. 2004; Tsugeki et al. 2009). Recently, paleoecological researchers have started to use multiple regression and other statistical modeling approaches to investigate climatic impact on deep-sea ecosystems. For example, recent work has compared microfossil species diversity with independent proxies for temperature, surface productivity and seasonality of productivity, and using multiple regression and model averaging (Hunt et al. 2005; Yasuhara et al. 2009, 2012a,c). However, such approaches have seldom been applied to understanding human-induced ecological degradation research in marine systems.

Independent evidence of environmental history can be obtained by a variety of means. There are well-established methods for reconstructing concentrations of various heavy metals and organic pollutants, oxygen content, temperature, salinity, and pH using core sediments (Cronin et al. 2005; Kemp et al. 2005; Yasuhara and Yamazaki 2005; Pelejero et al. 2010), and stable isotopes and trace elements of microfossil shells allow inference of various environmental parameters (Cronin 2010). These data will be critical in attempts to assess the relative importance of climate versus eutrophication, or organic pollution versus metal pollution, in affecting the marine organisms.

### Other microfossil records indicative of ecological history

Sediment core records of fish scales are another rich archive of long-term ecological history. However, because fish population changes reconstructed from fish scale records tend to be interpreted as responses to natural climate changes (Field et al. 2009; Finney et al. 2010) we did not include these studies in this review of human-induced ecological degradation although future work may explore the relative importance of natural climate fluctuations versus overfishing through explicit statistical modeling. Similarly, our review excluded phytoplankton pigment records because of our focus on body fossils, rather than chemical remains. Pigment studies are rarer in marine systems compared with freshwater habitats (see Leavitt and Hodgson 2002), and they are concentrated in well-studied regions regarding microfossils such as Gulf of Mexico and Baltic Sea where their results have complemented those from more traditional microfossil studies (Reuss et al. 2005; Rabalais et al. 2007; Savage et al. 2010).

### Unexplored ecosystems

Microfossil-based paleoecological studies of marine ecological degradation are still largely limited to mid-latitude estuaries and embayments. There is almost no such research in tropical coral reefs and mangroves, in polar environments, or in the deep sea, in spite of the high vulnerability of these habitats and public concern about them. Such studies on open shelf communities are also limited in number compared with those on estuary and embayment communities (Table S1).

### Unexplored environmental issues

Ocean acidification is one of the most serious problems facing ocean life today, especially for organisms with calcified external skeletons (Orr et al. 2005; Hoegh-Guldberg et al. 2007; Fabry et al. 2008). Thus, calcareous microfossils such as foraminifera and ostracods are ideal for investigating the ecological impact of acidification over time. Micropaleontological research has revealed that glacial-interglacial changes in atmospheric carbon dioxide with the resulting changes in sea-water acidity have influenced marine calcification over the last 50,000 years (Barker and Elderfield 2002). However, culture experiments to determine the physiological effects of acidification on the organisms used in micropaleontological analyses have just begun (Spero et al. 1997; Kuroyanagi et al. 2009; Fujita et al. 2011).

## Conclusions

In this review, we analyzed patterns in published microfossil records to better understand spatiotemporal trends of marine ecological degradation across multiple scales, and to illustrate the usefulness of microfossils in detecting ongoing ecological changes during the Anthropocene (Zalasiewicz et al. 2008). The results indicated that: (1) ecological degradation in marine systems began significantly earlier in Europe and North America (∼1800s) compared with Asia (post-1900) due to earlier industrialization in European and North American countries, (2) ecological degradation accelerated globally in the late 20th century due to post-World War II economic and population growth and exponential increase in use of chemical fertilizers, (3) recovery from the degraded state in late 20th century after restoration efforts and environmental regulations were implemented only in limited localities. The predominant cause of degradation detected in microfossil records was nutrient enrichment and the resulting symptoms of eutrophication including hypoxia.

Degradation of ocean ecosystems has been characterized as the collapse of large animals and the rise of microbes (Jackson 2001; Jackson et al. 2001; Jackson 2008), with many nonresistant species becoming ecologically extinct and replaced by a few opportunistic or tolerant species. Microfossil records show similar processes evidenced by high-density and low-diversity assemblages in eutrophic coastal areas worldwide (Alve 1995; Tsujimoto et al. 2008).

Downcore microfossil studies are also important for an improved understanding of the natural baseline, which is often difficult to reconstruct (Pauly 1995; Jackson et al. 2001; Knowlton and Jackson 2008). Although microfossil-based human-induced ecological degradation research has often focus solely on the last few hundreds of years, longer records from the past thousands of years are needed to better understand the severity of ecological degradation relative to pre-impact baselines.

## Supporting Information

**Figure S1.** Published age distribution of papers compiled in this review. Data from Table S1.

**Figure S2**. Global distribution of marine ecological degradation in downcore microfossil records. (a) ostracod and (b) foraminiferal data sites (all of them are benthic, except one planktic foraminiferal data site from Santa Barbara Basin, USA: see Table S1). NA: site with no ostracod or foraminiferal data. Data from Table S1.

**Figure S3.** Global distribution of marine ecological degradation in downcore microfossil records. (a) diatom and (b) dinoflagellate data sites. NA: site with no diatom or dinoflagellate data. Data from Table S1.

**Figure S4.** Global distribution of marine ecological degradation caused by human-induced eutrophication. NA: site with no eutrophication evidence. Data from Table S1.

**Figure S5.** Chesapeake Bay paleoecological records in the core MD99-2209/RD-98. *Loxoconcha* sp. relative abundance record (A) and its closeup for the last 200 years (B); *Cytheromorpha curta* relative abundance record (C) and its closeup (D); and conceptual figure explaining core locations of deep-channel site of MD99-2209/RD-98 (This figure) and shallow, channel edge site (E: see text for detail). Data from Cronin et al. (2005) and Cronin (unpublished data).

**Table S1.** Dataset used for the present paper (see Supplementary Text for references). Ostracod: benthic ostracod; Foram: benthic foraminifera. Planktic foram: planktic foraminifera. Dino: dinoflagellate. ND: not detectable.

**Supplementary Text S1.** References cited in Table S1.
