# Supplementary figures and images for: Human-induced marine ecological degradation: micropaleontological perspectives
Source: Ecol Evol. 2012 Nov 15;2(12):3242–68. doi: 10.1002/ece3.425 (PMC3539015; doi:10.1002/ece3.425)

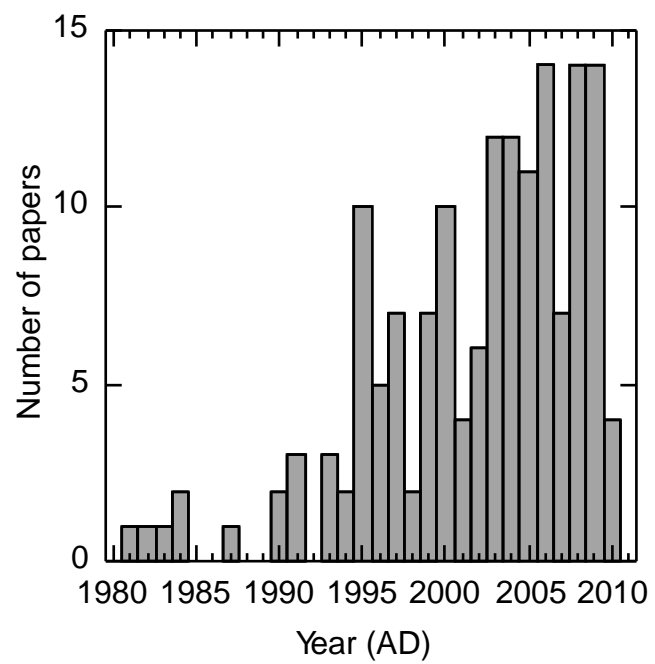

Fig. S1 Yasuhara et al.

Supplement: Supplementary file 1 [file ece30002-3242-SD1.pdf]

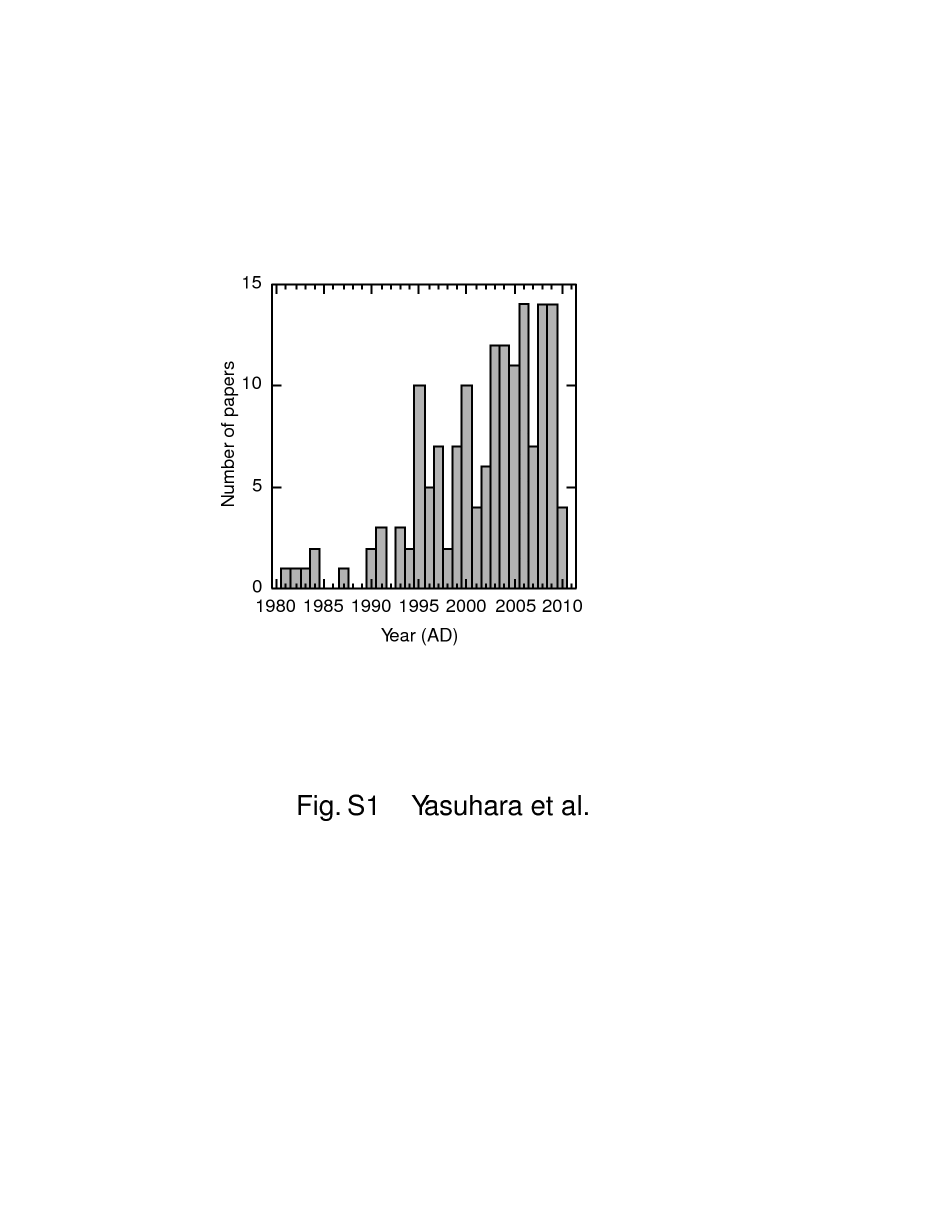

Supplement: Supplementary file 2 [file ece30002-3242-SD6.png]

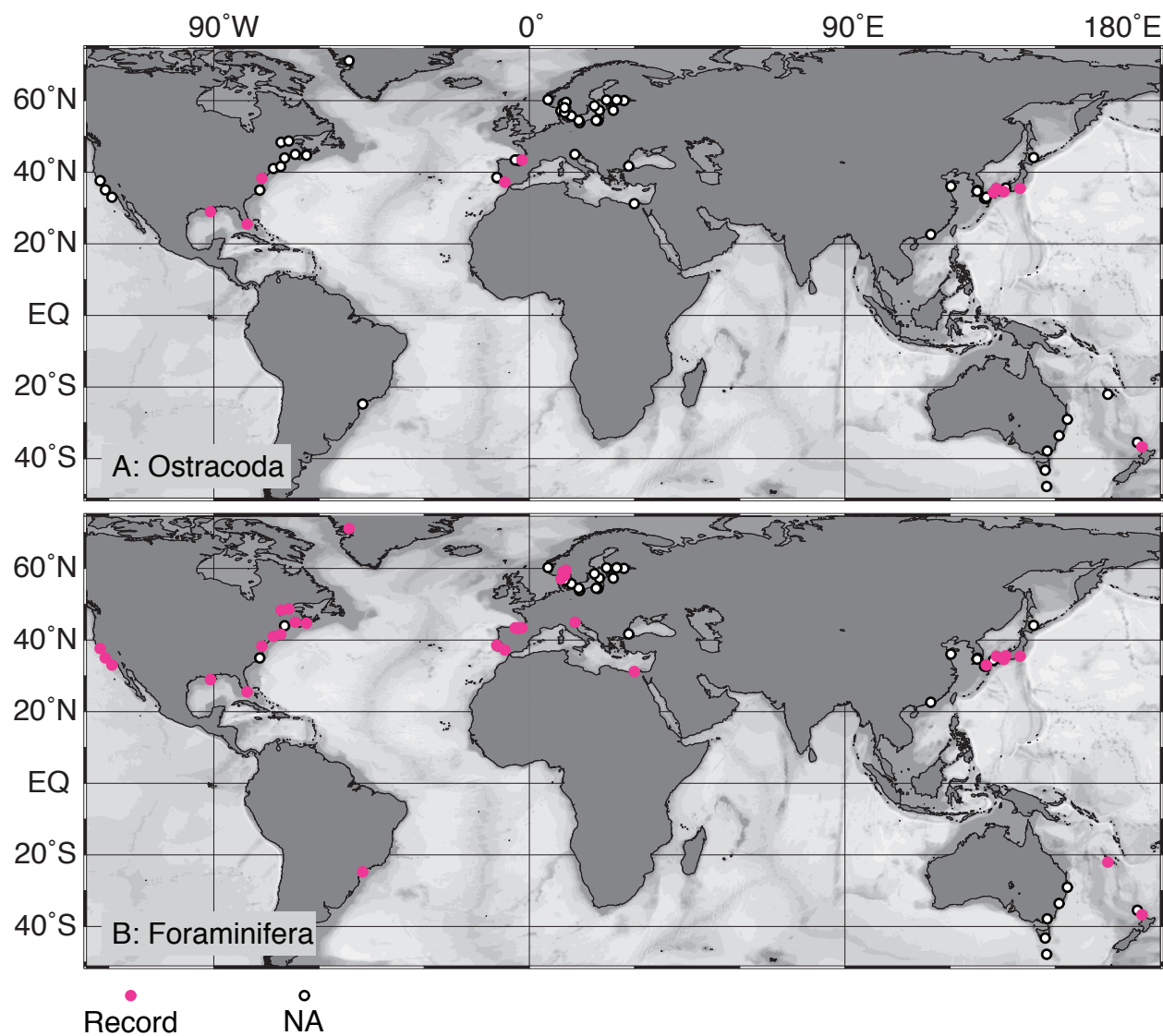

Fig. S2 Yasuhara et al.

Supplement: Supplementary file 4 [file ece30002-3242-SD2.pdf]

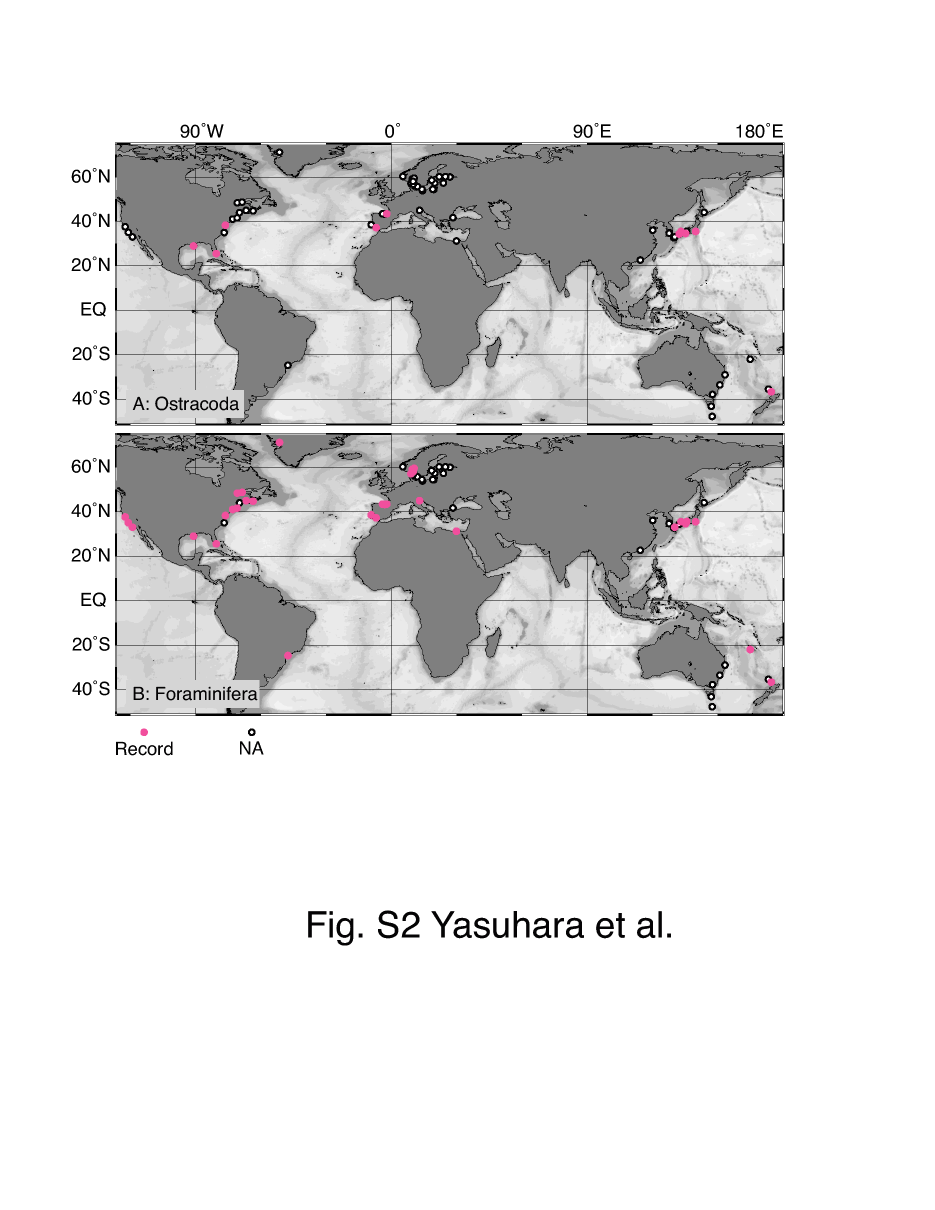

Supplement: Supplementary file 5 [file ece30002-3242-SD7.png]

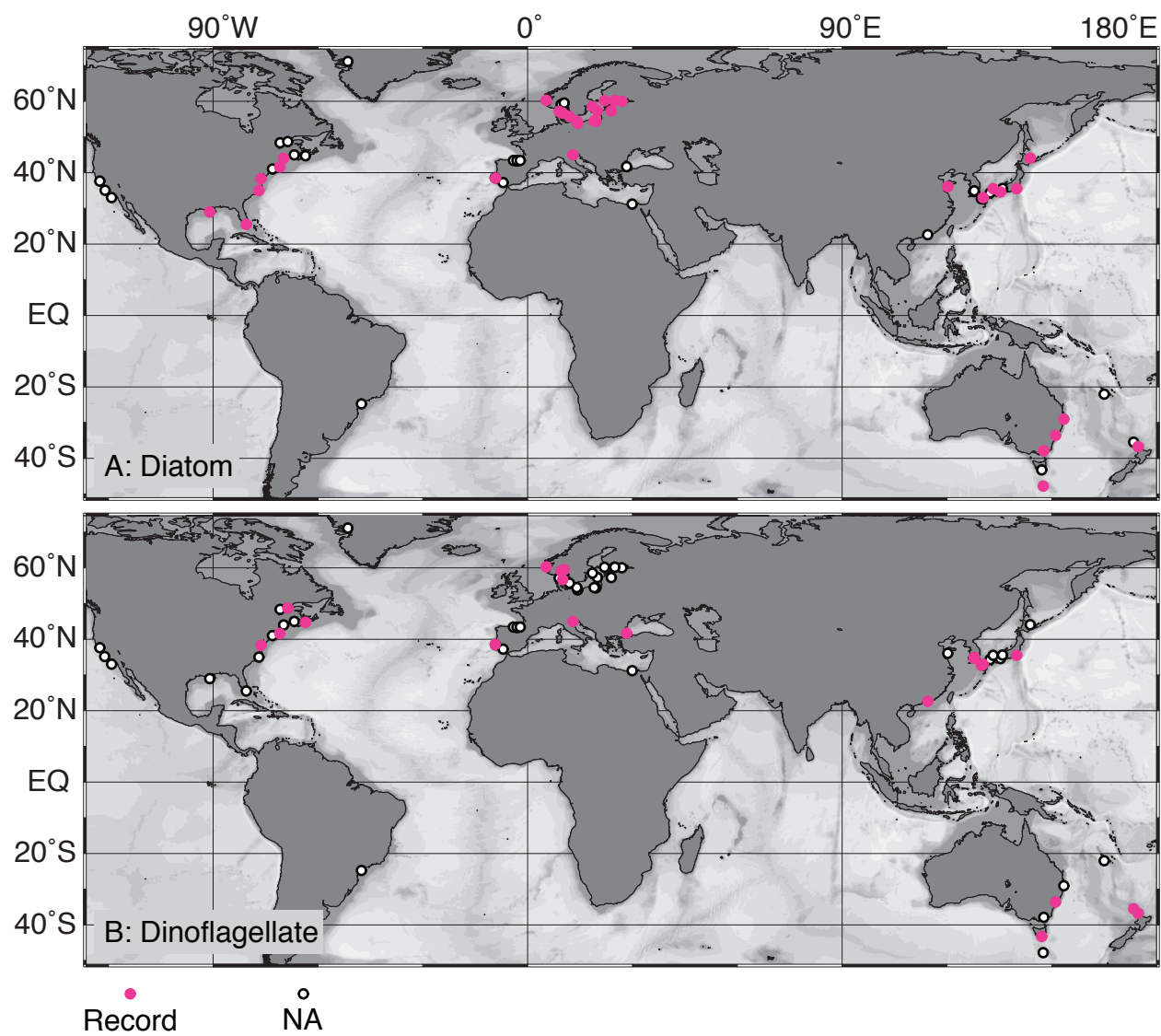

Fig. S3 Yasuhara et al.

Supplement: Supplementary file 7 [file ece30002-3242-SD3.pdf]

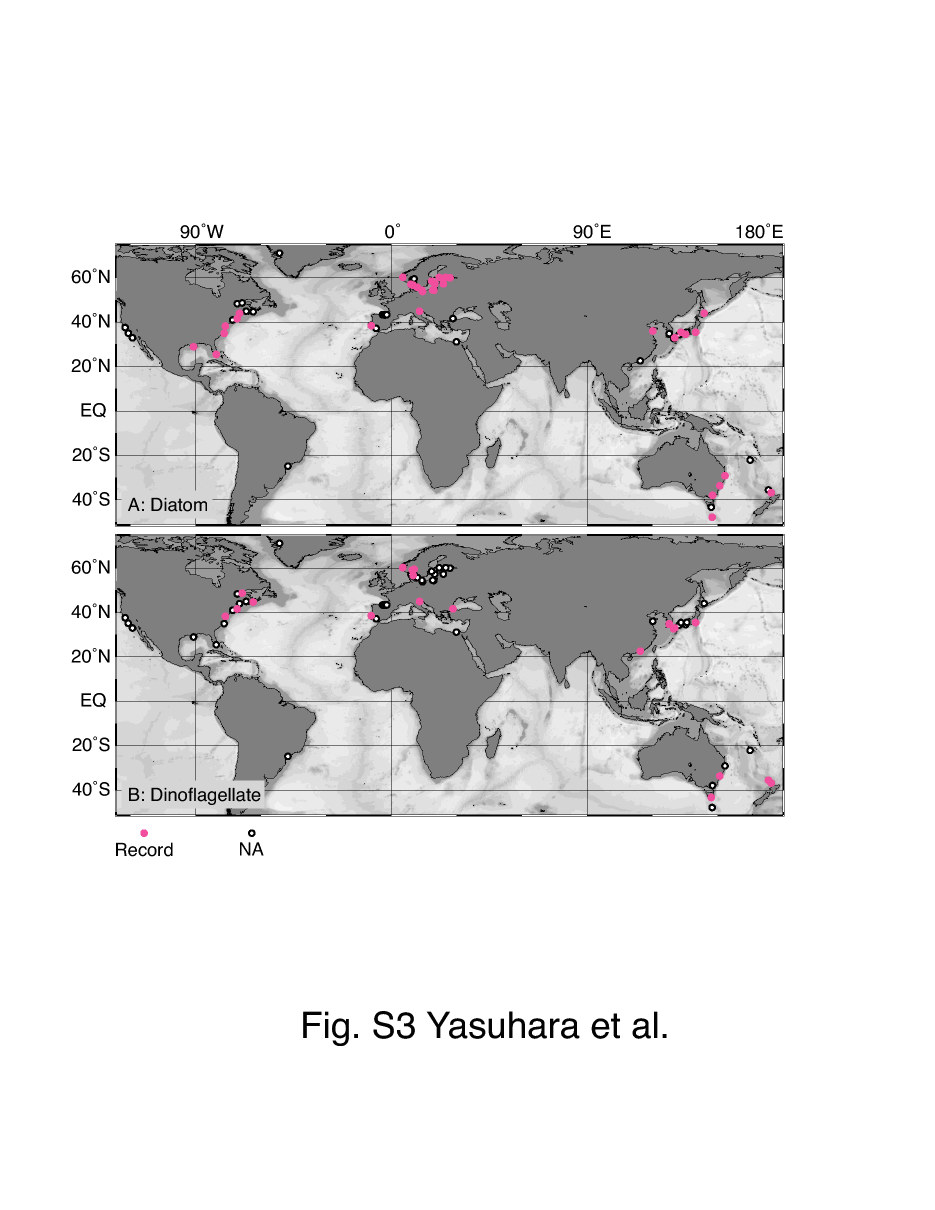

Supplement: Supplementary file 8 [file ece30002-3242-SD8.png]

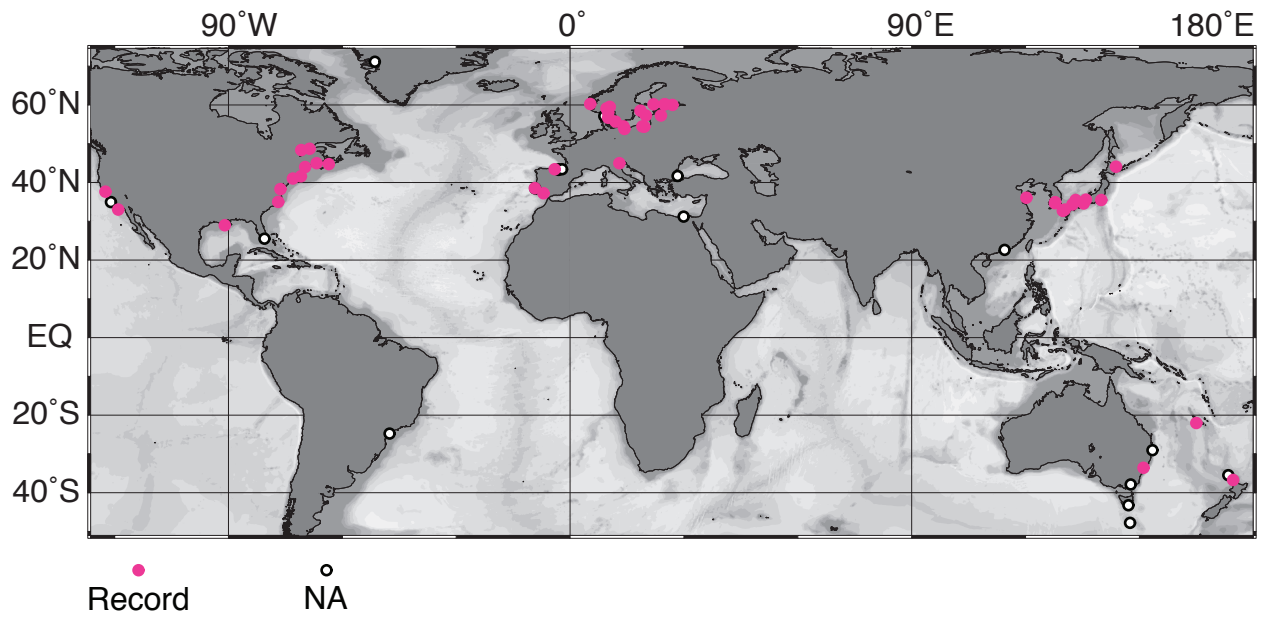

Fig. S4 Yasuhara et al.

Supplement: Supplementary file 10 [file ece30002-3242-SD4.pdf]

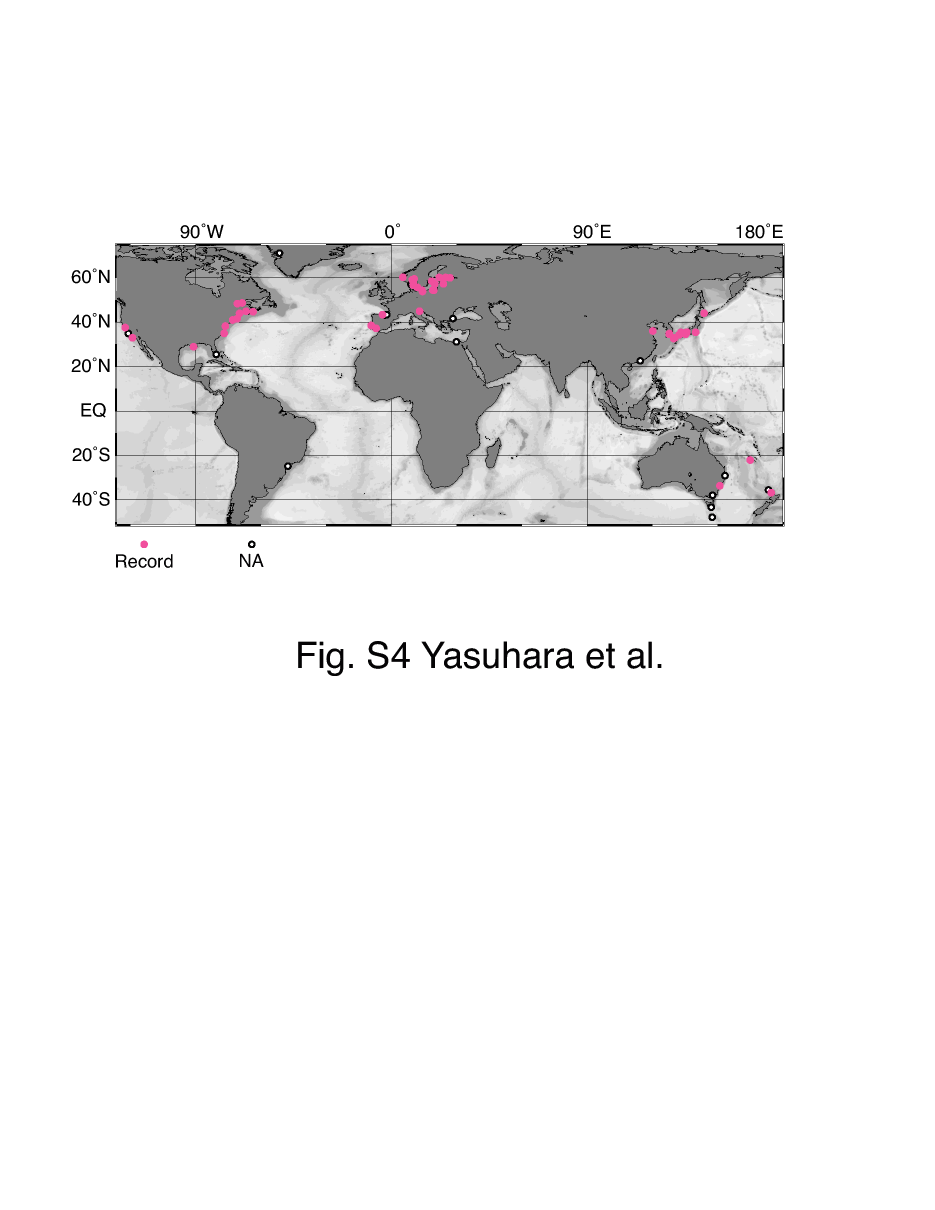

Supplement: Supplementary file 11 [file ece30002-3242-SD9.png]

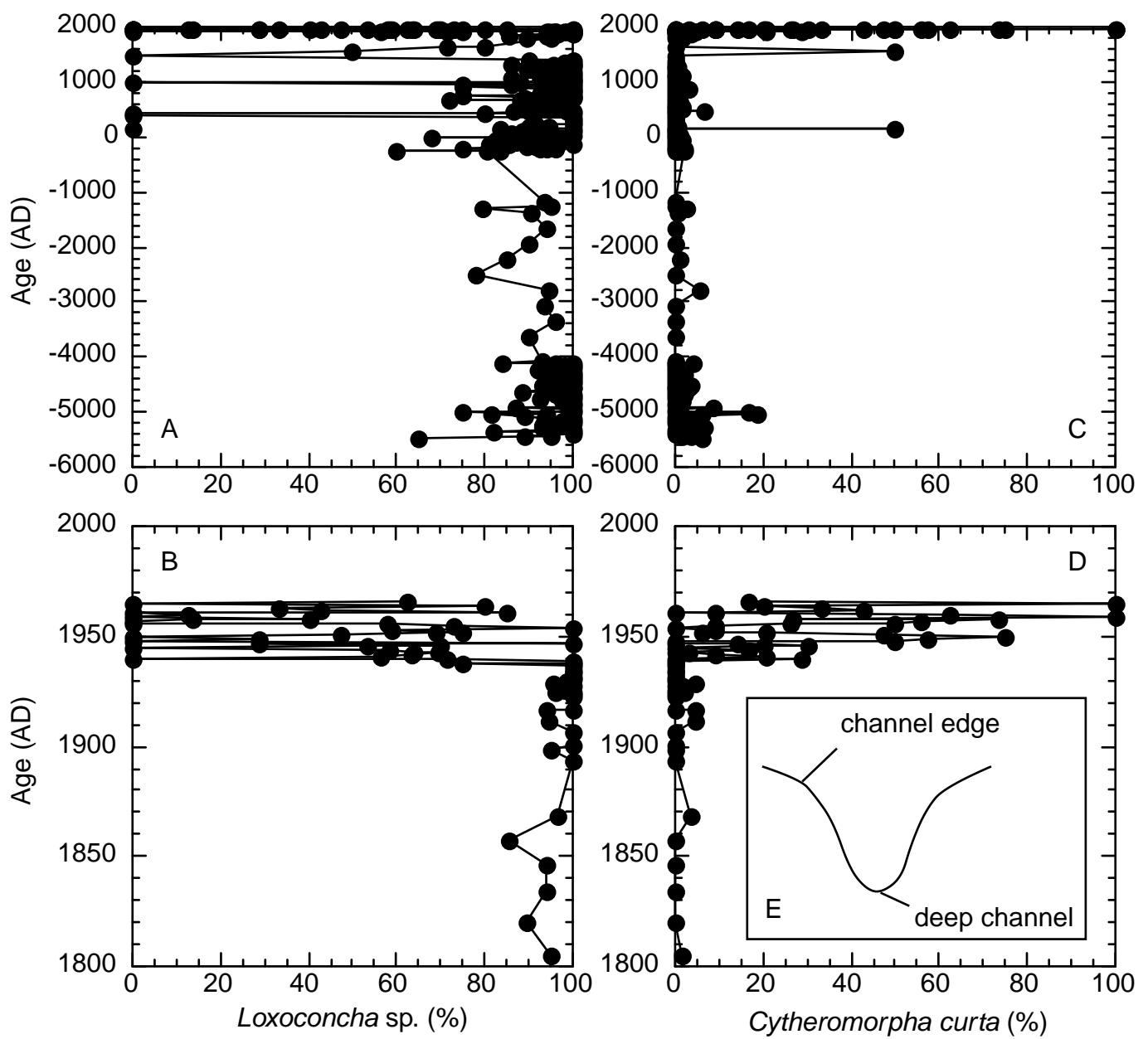

Fig. S5 Yasuhara et al.

Supplement: Supplementary file 13 [file ece30002-3242-SD5.pdf]

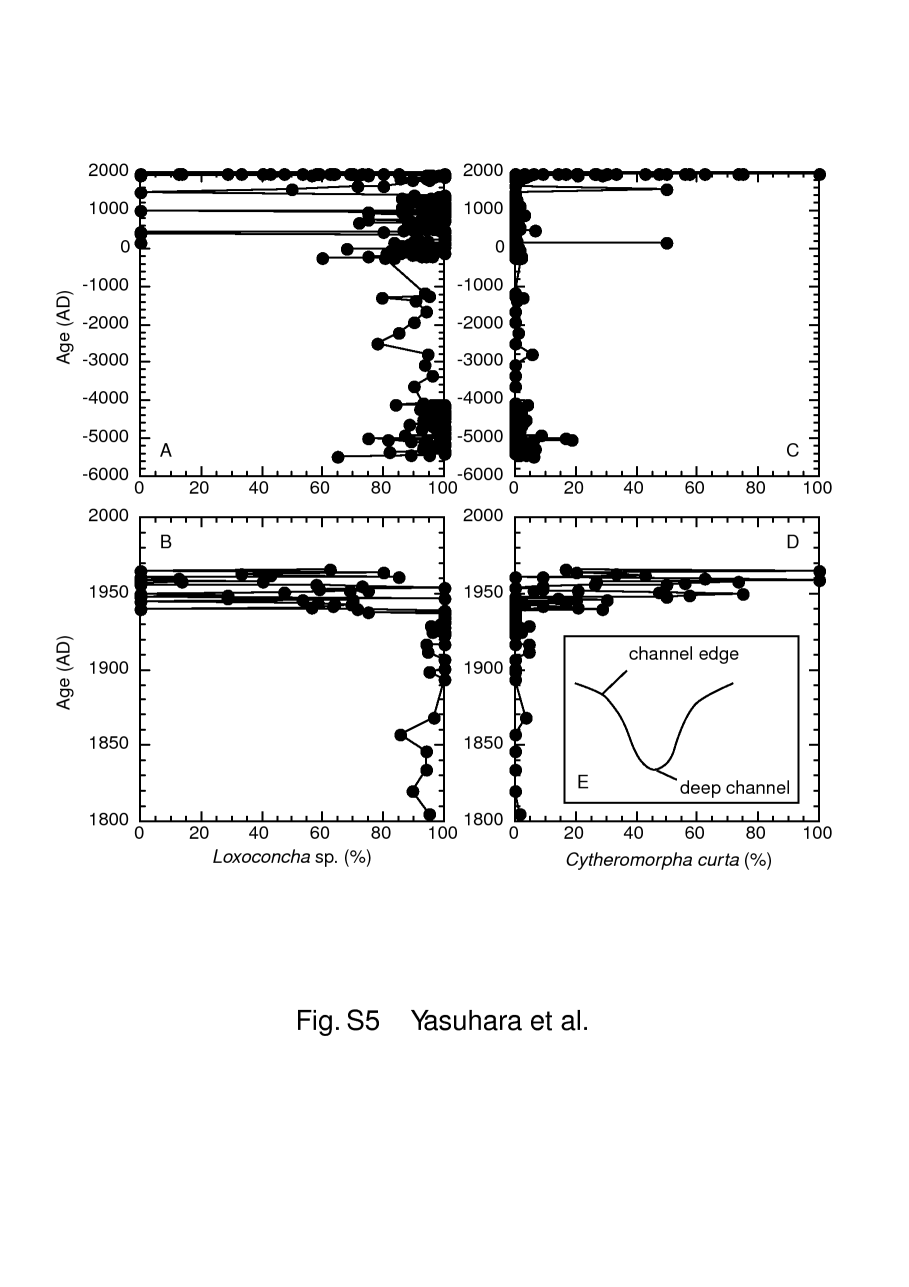

Supplement: Supplementary file 14 [file ece30002-3242-SD10.png]
