# Supplementary material for: Human-induced marine ecological degradation: micropaleontological perspectives
Source: Ecol Evol. 2012 Nov 15;2(12):3242–68. doi: 10.1002/ece3.425 (PMC3539015; doi:10.1002/ece3.425)
Supplement: Supplementary file 17 [file ece30002-3242-SD17.doc]

**References Cited in Table S1**

Akimoto, K., K. Nakamura, H. Kondo, H. Ishiga, and K. Dozen. 2004. Environmental reconstruction based on heavy metals, diatoms an benthic foraminifes in the Isahaya reclamation area, Nagasaki, Japan. Environmental Micropaleontology, Microbiology and Meiobenthology **1**:83–104.

Alvarez Zarikian, C. A., P. L. Blackwelder, T. Hood, T. A. Nelsen, and C. Featherstone. 2000. Ostracods as indicators of natural and anthropogenically-induced changes in coastal marine environments. Coasts at the Millennium, Proceedings of the 17th International Conference of The Coastal Society, Portland, OR USA, July 9-12:896–905.

Alvarez Zarikian, C. A., P. K. Swart, T. Hood, P. L. Blackwelder, T. A. Nelsen, and C. Featherstone. 2001. A century of environmental variability in Oyster Bay using ostracode ecological and isotopic data as paleoenvironmental tools. Bulletins of American Paleontology **361**:133-143.

Alve, E. 1991. Foraminifera, climatic change, and pollution: a study of late Holocene sediments in Drammensfjord, southeast Norway. Holocene **1**:243–261.

Alve, E. 1996. Benthic foraminiferal evidence of environmental change in the Skagerrak over the past six decades. Norges Geologiske Undersøkelse **430**:85–93.

Alve, E. 2000. Environmental stratigraphy: A case study reconstructing bottom water oxygen conditions in Frierfjord, Norway, over the past five centuries. Pages 324–350 *in* R. E. Martin, editor. Environmental Micropaleontology: The Application of Microfossils to Environmental Geology. Kluwer Academic/Plenum Publishers, New York.

Alve, E., A. Lepland, J. Magnusson, and K. Backer-Owe. 2009. Monitoring strategies for re-establishment of ecological reference conditions: Possibilities and limitations. Marine Pollution Bulletin **59**:297–310.

Alve, E., and J. W. Murray. 1995. Benthic foraminiferal distribution and abundance changes in Skagerrak surface sediments: 1937 (Hiiglund) and 1992/ 1993 data compared. Marine Micropaleontology **25**:269–288.

Amorim, A., and B. Dale. 2006. Historical cyst record as evidence for the recent introduction of the dinoflagellate *Gymnodinium* *catenatum* in the north-eastern Atlantic. African Journal of Marine Science **28**:193–197.

Amsinck, S. L., E. Jeppesen, and D. Ryves. 2003. Cladoceran stratigraphy in two shallow brackish lakes with special reference to changes in salinity, macrophyte abundance and fish predation. Journal of Paleolimnology **29**:495–507.

Andrén, E. 1999. Changes in the composition of the diatom flora during the last century indicate increased eutrophication of the Oder Estuary, south-western Baltic Sea. Estuarine, Coastal and Shelf Science **48**:665–676.

Andrén, E., T. Andrén, and H. Kunzendorf. 2000. Holocene history of the Baltic Sea as a background for assessing records of human impact in the sediments of the Gotland Basin. Holocene **10**:687–702.

Andrén, E., G. Shimmield, and T. Brand. 1999. Environmental changes of the last three centuries indicated by siliceous microfossil records from the southwestern Baltic Sea. Holocene **9**:25–38.

Bao, R., M. da Codnceição Freitas, and C. Andrade. 1999. Separating eustatic from local environmental effects: a late-Holocene record of coastal change in Albufeira Lagoon, Portugal. Holocene **9**:341–352.

Barmawidjaja, D. M., G. J. Vanderzwaan, F. J. Jorissen, and S. Puskaric. 1995. 150 years of eutrophication in the northern Adriatic Sea - evidence from a benthic foraminiferal record. Marine Geology **122**:367-384.

Bartels-Jónsdóttir, H. B., K. L. Knudsen, F. Abrantes, S. Lebreiro, and J. Eiriksson. 2006. Climate variability during the last 2000 years in the Tagus Prodelta, western Iberian Margin: Benthic foraminifera and stable isotopes. Marine Micropaleontology **59**:83–103.

Bartels-Jónsdóttir, H. B., A. H. L. Voelker, K. L. Knudsen, and F. Abrantes. 2009. Twentieth-century warming and hydrographical changes in the Tagus Prodelta, eastern North Atlantic. Holocene **19**:369–380.

Bernasconi, M. P., R. Melis, and D. J. Stanley. 2006. Benthic biofacies to interpret Holocene environmental changes and human impact in Alexandria's Eastern Harbour, Egypt. Holocene **16**:1163–1176.

Blackwelder, P., T. Hood, C. Alvarez-Zarikian, T. A. Nelsen, and B. McKee. 1996. Benthic foraminifera from the NECOP study area impacted by the Mississippi River plume and seasonal hypoxia. Quaternary International **31**:19–36.

Brewster-Wingard, G. L., and S. E. Ishman. 1999. Historical trends in salinity and substrate in central Florida Bay: A paleoecological reconstruction using modern analogue data. Estuaries **22**:369–383.

Brunner, C. A., J. M. Beall, S. J. Bentley, and Y. Furukawa. 2006. Hypoxia hotspots in the Mississippi Bight. Journal of Foraminiferal Research **36**:95–107.

Brush, G. S. 2009. Historical land use, nitrogen, and coastal eutrophication: a paleoecological perspective. Estuaries and Coasts **32**:18–28.

Brush, G. S., and F. W. Davis. 1984. Stratigraphic evidence of human disturbance in an estuary. Quaternary Research **22**:91–108.

Brush, G. S., and W. B. Hilgartner. 2000. Paleoecology of submerged macrophytes in the upper Chesapeake Bay. Ecological Monographs **70**:645–667.

Cearreta, A., M. J. Irabien, E. Leorri, I. Yusta, I. W. Croudace, and A. B. Cundy. 2000. Recent anthropogenic impacts on the Bilbao estuary, Northern Spain: Geochemical and microfaunal evidence. Estuarine, Coastal and Shelf Science **50**:571–592.

Cearreta, A., M. J. Irabien, E. Leorri, I. Yusta, A. Quintanilla, and A. Zabaleta. 2002a. Environmental transformation of the Bilbao estuary, N. Spain: microfaunal and geochemical proxies in the recent sedimentary record. Marine Pollution Bulletin **44**:487–503.

Cearreta, A., M. J. Irabien, I. Ulibarri, I. Yusta, I. W. Croudace, and A. B. Cundy. 2002b. Recent salt marsh development and natural regeneration of reclaimed areas in the Plentzia Estuary, N. Spain. Estuarine, Coastal and Shelf Science **54**:863–886.

Chmura, G. L., A. Santos, V. Pospelova, Z. Spasojevic, R. Lam, and J. S. Latimer. 2004. Response of three paleo-primary production proxy measures to development of an urban estuary. Science of the Total Environment **320**:225–243.

Clarke, A., S. Juggins, and D. Conley. 2003. A 150-year reconstruction of the history of coastal eutrophication in Roskilde Fjord, Denmark. Marine Pollution Bulletin **46**:1615–1629.

Clarke, A. L., K. Weckstrom, D. J. Conley, N. J. Anderson, F. Adser, E. Andren, V. N. de Jonge, M. Ellegaard, S. Juggins, P. Kauppila, A. Korhola, N. Reuss, R. J. Telford, and S. Vaalgamaa. 2006. Long-term trends in eutrophication and nutrients in the coastal zone. Limnology and Oceanography **51**:385–397.

Cooper, S. R. 1995. Chesapeake Bay watershed historical land use: impact on water quality and diatom communities. Ecological Applications **5**:703–723.

Cooper, S. R., and G. S. Brush. 1991. Long-term history of Chesapeake Bay anoxia. Science **254**:992–996.

Cooper, S. R., and G. S. Brush. 1993. A 2,500-year history of anoxia and eutrophication in Chesapeake Bay. Estuaries **16**:617–626.

Cooper, S. R., S. K. McGlothlin, M. Madritch, and D. L. Jones. 2004. Paleoecological evidence of human impacts on the Neuse and Pamlico estuaries of North Carolina, USA. Estuaries **27**:617–633.

Cronin, T. M., and C. D. Vann. 2003. The sedimentary record of climatic and anthropogenic influence on the Patuxent estuary and Chesapeake Bay ecosystems. Estuaries **26**:196-209.

Dale, B. 2000. Dinoflagellate cysts as indicators pf cultural eutrophication and industrial pollution in coastal sediments. Pages 305–321 *in* R. E. Martin, editor. Environmental Micropaleontology: The Application of Microfossils to Environmental Geology. Kluwer Academic/Plenum Publishers, New York.

Dale, B., and A. Fjellså. 1994. Dinoflagellate cysts as paleoproductivity indicators: state of the art, potential and limits. Pages 521–537 *in* R. Zahn, M. A. Kaminski, L. Labeyrie, and T. F. Pederson, editors. Carbon Cycling in the Glacial Ocean: Constraints on the Ocean’s Role in Global Change. Springer-Verlag, Berlin.

Dale, B., T. A. Thorsen, and A. Fjellså. 1999. Dinoflagellate cysts as indicators of cultural eutrophication in the Oslofjord, Norway. Estuarine, Coastal and Shelf Science **48**:371–382.

de Mahiques, M. M., L. Burone, R. C. L. Figueira, A. A. de Oliveira Lavenére-Wanderley, B. Capellari, C. E. Rogacheski, C. P. Barroso, L. A. S. dos Santos, L. M. Cordero, and M. C. Cussioli. 2009. Anthropogenic influences in a lagoonal environment: a multiproxy approach at the valo grande mouth, Cananéia-Iguape system (SE Brazil). Brazilian Journal of Oceanography **57**:325–337.

Debenay, J. P., and J. M. Fernandez. 2009. Benthic foraminifera records of complex anthropogenic environmental changes combined with geochemical data in a tropical bay of New Caledonia (SW Pacific). Marine Pollution Bulletin **59**:311–322.

Elberling, B., K. L. Knudsen, P. H. Kristensen, and G. Asmund. 2003. Applying foraminiferal stratigraphy as a biomarker for heavy metal contamination and mining impact in a fiord in West Greenland. Marine Environmental Research **55**:235-256.

Ellegaard, M., A. L. Clarke, N. Reuss, S. Drew, K. Weckström, S. Juggins, N. J. Anderson, and D. J. Conley. 2006. Multi-proxy evidence of long-term changes in ecosystem structure in a Danish marine estuary, linked to increased nutrient loading. Estuarine, Coastal and Shelf Science **68**:567–578.

Field, D. B., T. R. Baumgartner, C. D. Charles, V. Ferreira-Bartrina, and M. D. Ohman. 2006. Planktonic foraminifera of the California Current reflect 20th-century warming. Science **311**:63–66.

Gonzalez-Regalado, M. L., F. Ruiz, J. I. Baceta, E. González-Regalado, and J. M. Munoz. 2001. Total benthic foraminifera assemblages in the southwestern Spanish estuaries. Geobios **34**:39–51.

González-Regalado, M. L., F. Ruiz, and J. Borrego. 1996. Evolución de la distribución de los foraminíferos bentónicos en un medio contaminado: el estuario del río Odiel (Huelva, S.O. de España). Revista Española de Paleontología **11**:1–10.

Grenfell, H. R., B. W. Hayward, and M. Horrocks. 2007. Foraminiferal record of ecological impact of deforestation and oyster farms, Mahurangi Harbour, New Zealand. Marine and Freshwater Research **58**:475–491.

Grönlund, T. 1993. Diatoms in surface sediments of the Gotland Basin in the Baltic Sea. Hydrobiologia **269-270**:235–242.

Hass, H. C. 1997a. The benthic foraminiferal response to late Holocene climate change over northern Europe. Grzybowsky Foundation Special Publication **5**:199–216.

Hass, H. C. 1997b. Recent and subrecent agglutinated foraminifera in four box cores from the Skagerrak (NE North Sea). Grzybowsky Foundation Special Publication **5**:217–226.

Hayward, B. H., H. R. Grenfell, A. T. Sabaa, and M. S. Morley. 2008. Ecological impact of the introduction to New Zealand of Asian date mussels and cordgrass—The foraminiferal, ostracod and molluscan record. Estuaries and Coasts **31**:941–959.

Hayward, B. H., H. R. Grenfell, A. T. Sabaa, M. S. Morley, and M. Horrocks. 2006. Effect and timing of increased freshwater runoff into sheltered harbor environments around Auckland City, New Zeland. Estuaries and Coasts **29**:165–182.

Hayward, B. W., H. R. Grenfell, K. Nicholson, R. Parker, J. Wilmhurst, M. Horrocks, A. Swales, and A. T. Sabaa. 2004. Foraminiferal record of human impact on intertidal estuarine environments in New Zealand’s largest city. Marine Micropaleontology **53**:37–66.

Hirose, K., M. Yasuhara, A. Tsujimoto, H. Yamazaki, and S. Yoshikawa. 2008. The succession of diatom assemblages and anthropogenically-induced environmental changes over the last 120 years, Osaka Bay, Japan. The Quaternary Research (Daiyonki-kenkyu) **47**:287–296.

Huvane, J. K., and S. R. Cooper. 2001. Diatoms as indicators of environmental change in sediment core from northeastern Florida Bay. Bulletins of American Paleontology **361**:145–158.

Ikeya, N. 1995. Ostracoda in sediment cores from Yokohama Port. Changes in marine organisms and environments at Yokohama Port: research on fossils in sediment core samples, Yokohama Environmental Research Institute, Report no. 116:27–33.

Irabien, M. J., A. Cearreta, E. Leorri, J. Gómez, and J. Viguri. 2008. A 130 year record of pollution in the Suances estuary (southern Bay of Biscay): Implications for environmental management. Marine Pollution Bulletin **56**:1719–1727.

Irizuki, T., Y. Nakamura, K. Takayasu, and S. Sakai. 2003. Faunal changes in Ostracoda (Crustacea) in Lake Nakaumi, southwest Japan, over the last 40 years. Geoscience Report of Shimane University **22**:149–160.

Irwin, A., G. M. Mallegraeff, A. McMinn, J. Harrison, and H. Heijnis. 2003. Cyst and radionuclide evidence demonstrate historic *Gymnodinium* *catenatum* dinoflagellate populations in Manukau and Hokianga Harbours, New Zealand. Harmful Algae **2**:61–74.

Ishman, S. E. 2000. Benthic foraminiferal distributions in South Florida: Analogues to historical changes. Pages 371–383 *in* R. E. Martin, editor. Environmental Micropaleontology: The Application of Microfossils to Environmental Geology. Kluwer Academic/Plenum Publishers, New York.

Ishman, S. E., T. M. Cronin, G. L. Brewster-Wingard, D. A. Willard, and D. J. Verardo. 1998. A record of ecosystem change, Manatee Bay, Bay, Barnes Sound, Florida. Journal of Coastal Research **26**:125–138.

Jankowska, D., M. Witak, and D. Huszczo. 2005. Paleoecological changes of the Vistula Lagoon in the last 7,000 YBP based on diatom flora. Oceanological and Hydrobiological Studies **34**:109–129.

Karlsen, A. W., T. M. Cronin, S. E. Ishman, D. A. Willard, R. Kerhin, C. W. Holmes, and M. Marot. 2000. Historical trends in Chesapeake Bay dissolved oxygen based on benthic foraminifera from sediment cores. Estuaries **23**:488–508.

Katsuki, K., Y. Miyamoto, K. Yamada, H. Takata, K. Yamaguchi, N. D., H. Coops, H. Kunii, R. Nomura, and B. K. Khim. 2008. Eutrophication-induced changes in Lake Nakaumi, southwest Japan. Journal of Paleolimnology **40**:1115–1125.

Katsuki, K., K. Seto, R. Nomura, K. Maekawa, and B. K. Khim. 2009. Effect of human activity on Lake Saroma (Japan) during the past 150 years: Evidence by variation of diatom assemblages. Estuarine, Coastal and Shelf Science **81**:215–224.

Kauppila, P., K. Weckström, S. Vaalgamaa, A. Korhola, H. Pitkänen, N. Reuss, and S. Drew. 2005. Tracing pollution and recovery using sediments in an urban estuary, northern Baltic Sea: are we far from ecological reference conditions? Marine Ecology Progress Series **290**:35–53.

Kim, H. S., and K. Matsuoka. 1998. Process of eutrophication estimated by dinoflagellate cyst assemblages in Omura Bay, Kyushu, West Japan. Bulletin of the Plankton Society of Japan **45**:133–147.

Kim, S. Y., C. H. Moon, H. J. Cho, and D. I. Lim. 2009. Dinoflagellate cysts in coastal sediments as indicators of eutrophication: A case of Gwangyang Bay, South Sea of Korea. Estuaries and Coasts **32**:1225–1233.

Korhola, A., and T. Blom. 1996. Marked early 20th century pollution and the subsequent recovery of Töölö Bay, central Helsinki, as indicated by subfossil diatom assemblage changes. Hydrobiologia **341**:169–179.

Köster, D., J. Lichter, P. D. Lea, and A. Nurse. 2007. Historical eutrophication in a river-estuary complex in mid-coast Maine. Ecological Applications **17**:765–778.

Leśniewska, M., and M. Witak. 2008. Holocene diatom biostratigraphy of the SW Gulf of Gdańsk, Southern Baltic Sea (part III). Oceanological and Hydrobiological Studies **37**:35–52.

Liu, D. Y., J. Sun, J. Zhang, and G. S. Liu. 2008. Response of the diatom flora in Jiaozhou Bay, China to environmental changes during the last century. Marine Micropaleontology **66**:279–290.

Marret, F., P. Mudie, A. Aksu, and R. N. Hiscott. 2009. A Holocene dinocyst record of a two-step transformation of the Neoeuxinian brackish water lake into the Black Sea. Quaternary International **197**:72–86.

Matsumoto, E. 1981. Research on coastal marine pollution. Chishitsu News **319**:52–58.

Matsumoto, E. 1983. Environmental changes recorded in sediments of coastal marine zone closed by big city. The Memoirs of the Geological Society of Japan **23**:91–95.

Matsumoto, E., and Y. Saito. 1984. Environmental changes of Tokyo Bay by man in comparison with environmental changes by nature during geological history. Bulletin of the Geological Survey of Japan **35**:243–260.

Matsuoka, K. 1995. Dinoflagellate cyst assemblage in core samples of Yokohama Port St. 1. Changes in marine organisms and environments at Yokohama Port: research on fossils in sediment core samples, Yokohama Environmental Research Institute, Report no. 116:45–61.

Matsuoka, K. 1999. Eutrophication process recorded in dinoflagellate cyst assemblages — a case of Yokohama Port, Tokyo Bay, Japan. Science of the Total Environment **231**:17–35.

Matsuoka, K. 2001. Further evidence for a marine dinoflagellate cyst as an indicator of eutrophication in Yokohama Port, Tokyo Bay, Japan: Comments on a discussion by B. Dale. Science of the Total Environment **264**:221–233.

Matsuoka, K. 2004. Change in the aquatic environment of Isahaya Bay, Ariake Sound, West Japan: From the view point of dinoflagellate cyst assemblage. Bulletin on Coastal Oceanography **42**:55–59.

Matsuoka, K., and H. S. Kim. 1999. Process of eutrophication in enclosed seas recorded in dinoflagellate cyst assemblages and sediments – the case in Nagasaki Bay, west Japan –. Fossils (Kaseki) **66**:1–15.

Matsuoka, K., and H. H. Shin. 2010. Environmental changes in the inner part of Ariake Sound, west Japan recorded in dinoflagellate cyst assemblages. Pages 111–120 *in* A. Ishimatsu and H.-J. Lie, editors. Coastal Environmental and Ecosystem Issues of the East China Sea. TERRAPUB and Nagasaki University, Tokyo and Nagasaki.

Matthews, A., H. R. Grenfell, B. W. Hayward, and M. Horrocks. 2005. Foraminiferal record of sewage outfall impacts on the inner Manukau Harbour, Auckland, New Zealand. New Zealand Journal of Marine and Freshwater Research **39**:193–215.

McGann, M. 2008. High-resolution foraminiferal, isotopic, and trace element records from Holocene estuarine deposits of San Francisco Bay, California. Journal of Coastal Research **24**:1092–1109.

McGann, M. 2009. Review of impacts of contaminated sediment on microfaunal communities in the Southern California Bight. The Geological Society of America Special Paper **454**:413–455.

McGann, M., C. R. Alexander, and S. M. Bay. 2003. Response of benthic foraminifers to sewage discharge and remediation in Santa Monica Bay, California. Marine Environmental Research **56**:299–342.

McMinn, A., G. M. Hallegraeff, P. Thomson, A. V. Jenkinson, and H. Heijnis. 1997. Cyst and radionucleotide evidence for the recent introduction of the toxic dinoflagellate *Gymnodinium* *catenatum* into Tasmanian waters. Marine Ecology Progress Series **161**:165–172.

McMinn, A., H. Heijnis, A. Murray, and G. Hallegraeff. 2004. Diatom and dinoflagellate assemblages of the Hawkesbury River, N.S.W., over the last two centuries: evidence for changes in hydrology. Alcheringa **28**:505–514.

Miller, A. A. L., P. J. Mudie, and D. B. Scott. 1982. Holocene history of Bedford Basin, Nova Scotia: foraminifera, dinoflagellate, and pollen records. Canadian Journal of Earth Sciences **19**:2342–2367.

Miller, U., and J. Risberg. 1990. Environmental changes, mainly eutrophication, as recorded by fossil siliceous micro-algae in two cores from the uppermost sediments of the north-western Baltic. Beiheft zur Nova Hedwigia **100**:237–253.

Moodley, L., S. R. Troelstra, and T. C. E. Van Weering. 1993. Benthic foraminiferal response to environmental change in the Skagerrak, northeastern North Sea. Sarsia **78**:129–139.

Nagy, J., and E. Alve. 1987. Temporal changes in foraminiferal faunas and impact of pollution in Sandebukta, Oslo Fjord. Marine Micropaleontology **12**:109–128.

Nelsen, T. A., P. Blackwelder, T. Hood, B. McKee, N. Romer, C. Alvarez-Zarikian, and S. Metz. 1994. Time-based correlation of biogenic, lithogenic and authigenic sediment components with anthropogenic inputs in the Gulf of Mexico NECOP study area. Estuaries **17**:873–885.

Nomura, R. 2003. Assessing the roles of artificial vs. natural impacts on brackish lake environments: foraminiferal evidence from Lake Nakaumi, southwestern Japan. Journal of the Geological Society of Japan **109**:197–214.

Nomura, R., and S. Kawano. 2011. Foraminiferal assemblages response to anthropogenic influence and parallel to decadal sea-level changes over the last 70 years in Lake Kugushi, Fukui Prefecture, southwest Japan. Quaternaly International **230**:44–56.

Nomura, R., and K. Seto. 2002. Influence of man-made construction on environmental conditions in brackish Lake Nakaumi, southwest Japan: Foraminiferal evidence. Journal of the Geological Society of Japan **108**:394–409.

Olli, K., A. Clarke, Å. Danielsson, J. Aigars, D. J. Conley, and T. Tamminen. 2008. Diatom stratigraphy and long-term dissolved silica concentrations in the Baltic Sea. Journal of Marine Systems **73**:284–299.

Osterman, L. E., R. Z. Poore, and P. W. Swarzenski. 2008. The last 1000 years of natural and anthropogenic low-oxygen bottom-water on the Louisiana shelf, Gulf of Mexico. Marine Micropaleontology **66**:291–303.

Osterman, L. E., R. Z. Poore, P. W. Swarzenski, D. B. Senn, and S. F. DiMarco. 2009. The 20th-century development and expansion of Louisiana shelf hypoxia, Gulf of Mexico. Geo-Marine Letters **29**:405–414.

Osterman, L. E., R. Z. Poore, P. W. Swarzenski, and R. E. Turner. 2005. Reconstructing a 180 yr record of natural and anthropogenic induced low-oxygen conditions from Louisiana continental shelf sediments. Geology **33**:329–332.

Paetzel, M., and H. Schrader. 1995. Sewage history in the anoxic sediments of the fjord Nordåsvannet, western Norway (II): The origin of the sedimented organic matter fraction. Norsk Geologisk Tidsskrift **75**:146–155.

Parsons, M. L., and Q. Dortch. 2002. Sedimentological evidence of an increase in *Pseudo-nitzschia* (Bacillariophyceae) abundance in response to coastal eutrophication. Limnology and Oceanography **47**:551–558.

Parsons, M. L., Q. Dortch, R. E. Turner, and N. R. Rabalais. 2006. Reconstructing the development of eutrophication in Louisiana salt marshes. Limnology and Oceanography **51**:524–544.

Pascual, A., J. Rodriguez-Lazaro, O. Weber, and J. M. Jouanneau. 2002. Late Holocene pollution in the Gernika estuary (southern Bay of Biscay) evidenced by the study of Foraminifera and Ostracoda. Hydrobiologia **475**:477-491.

Platon, E., B. K. Sen Gupta, N. N. Rabalais, and R. E. Turner. 2005. Effect of seasonal hypoxia on the benthic foraminiferal community of the Louisiana inner continental shelf. The 20th century record. Marine Micropaleontology **54**:263-283.

Pospelova, V., G. L. Chmura, W. S. Boothman, and J. S. Latimer. 2002. Dinoflagellate cyst records and human disturbance in two neighboring estuaries, New Bedford Harbor and Apponagansett Bay, Massachusetts (USA). Science of the Total Environment **298**:81–102.

Puskaric, S., G. W. Berger, and F. J. Jorissen. 1990. Successive appearance of subfossil phytoplankton species in Holocene sediments of the northern Adriatic and its relation to the increased eutrophication pressure. Estuarine, Coastal and Shelf Science **31**:177–187.

Rabalais, N. N., R. E. Turner, B. K. Sen Gupta, E. Platon, and M. L. Parsons. 2007. Sediments tell the history of eutrophication and hypoxia in the northern Gulf of Mexico. Ecological Applications **17**:S129–S143.

Ruiz, F., J. Borrego, M. L. González-Regalado, N. López González, B. Carro, and M. Abad. 2008. Impact of millennial mining activities on sediments and microfauna of the Tinto River estuary (SW Spain). Marine Pollution Bulletin **56**:1258–1264.

Ruiz, F., J. Borrego, M. L. González-Regalado, N. López-González, B. Carro, and M. Abad. 2009. Interaction between sedimentary processes, historical pollution and microfauna in the Tinto Estuary (SW Spain). Environmental Geology **58**:779–783.

Ruiz, F., M. L. González-Regalado, J. Borrego, M. Abad, and J. G. Pendón. 2004. Ostracoda and Foraminifera as short-term tracers of environmental changes in very polluted areas: the Odiel Estuary (SW Spain). Environmental Pollution **129**:49–61.

Ruiz Muñoz, F., M. L. González-Regalado, J. Borrego Flores, and J. A. Morales. 1997. The response of ostracod assemblages to recent pollution and sedimentary processes in the Huelva Estuary, SW Spain. Science of the Total Environment **207**:91–103.

Ryves, D., A. L. Clarke, P. G. Appleby, S. L. Amsinck, E. Jeppesen, F. Landkildehus, and N. J. Anderson. 2004. Reconstructing the salinity and environment of the Limfjord and Vejlerne Nature Reserve, Denmark, using a diatom model for brackish lakes and fjords. Canadian Journal of Fisheries and Aquatic Sciences **61**:1988–2006.

Sætre, M. M. L., B. Dale, M. I. Abdullah, and G. P. Sætre. 1997. Dinoflagellate cysts as potential indicators of industrial pollution in a Norwegian Fjord. Marine Environmental Research **44**:167–189.

Sangiorgi, F., and T. H. Donders. 2004. Reconstructing 150 years of eutrophication in the north-western Adriatic Sea (Italy) using dinoflagellate cysts, pollen and spores. Estuarine, Coastal and Shelf Science **60**:69–79.

Sato, H. 1995. Diatom assemblages in core samples from Yokohama Port. Changes in marine organisms and environments at Yokohama Port: research on fossils in sediment core samples, Yokohama Environmental Research Institute, Report no. 116:63–76.

Saunders, K. M., D. A. Hodgson, J. Harrison, and A. McMinn. 2008. Palaeoecological tools for improving the management of coastal ecosystems: a case study from Lake King (Gippsland Lakes) Australia. Journal of Paleolimnology **40**:33–47.

Saunders, K. M., A. Mcminn, D. Roberts, D. A. Hodgson, and H. Heijnis. 2007. Recent human-induced salinity changes in Ramsar-listed Orielton Lagoon, south-east Tasmania, Australia: a new approach for coastal lagoon conservation and management. Aquatic Conservation: Marine and Freshwater Ecosystems **17**:51–70.

Saunders, K. M., and K. H. Taffs. 2009. Palaeoecology: A tool to improve the management of Australian estuaries. Journal of Environmental Management **90**:2730–2736.

Schafer, C. T., E. S. Collins, and J. N. Smith. 1991. Relationship of Foraminifera and thecamoebian distributions to sediments contaminated by pulp mill effluent: Saguenay Fiord, Quebec, Canada. Marine Micropaleontology **17**:255–283.

Scott, D. B., C. T. Schafer, C. Honig, and D. C. Younger. 1995. Temporal variations of benthic foraminiferal assemblages under or near aquaculture operations: documentation of impact history. Journal of Foraminiferal Research **25**:224–235.

Scott, D. B., R. Tobin, M. Williamson, F. S. Medioli, J. S. Latimer, W. A. Boothman, A. Asioli, and V. Haury. 2005. Pollution monitoring in two north American estuaries: Historical reconstructions using benthic foraminifera. Journal of Foraminiferal Research **35**:65–82.

Sen Gupta, B. K., R. E. Turner, and N. N. Rabalais. 1996. Seasonal oxygen depletion in continental-shelf waters of Louisiana: Historical record of benthic foraminifers. Geology **24**:227–230.

Shin, H. H., K. Mizushima, S. J. Oh, J. S. Park, II, H. Noh, M. Iwatani, K. Matsuoka, and Y. H. Yoon. 2010. Reconstruction of historical nutrient levels in Korean and Japanese coastal areas based on dinoflagellate cyst assemblages. Marine Pollution Bulletin **60**:1243–1258.

Swarzenski, P. W., P. L. Campbell, L. E. Osterman, and R. Z. Poore. 2008. A 1000-year sediment record of recurring hypoxia off the Mississippi River: The potential role of terrestrially-derived organic matter inputs. Marine Chemistry **109**:130–142.

Taffs, K. H., L. J. Farago, H. Heijnis, and G. Jacobsen. 2008. A diatom-based Holocene record of human impact from a coastal environment: Tuckean Swamp, eastern Australia. Journal of Paleolimnology **39**:71–82.

Tanimura, Y., M. Kato, C. Shimada, and E. Matsumoto. 2003. A one-hundred-year succession of planktonic and tychopelagic diatoms from 20th century Tokyo Bay. Bulletin of the National Science Museum, Tokyo. Series C, Geology & paleontology **29**:1–8.

Thibodeau, B., A. de Vernal, and A. Mucci. 2006. Recent eutrophication and consequent hypoxia in the bottom waters of the Lower St. Lawrence Estuary: Micropaleontological and geochemical evidence. Marine Geology **231**:37–50.

Thomas, E., I. Abramson, J. C. Varekamp, and M. R. Buchholtz ten Brink. 2004. Eutrophication of Long Island Sound as traced by benthic foraminifera. Proceedings 6th Biennual Long Island Sound Meeting (Groton, CT, October 2002):87–91.

Thomas, E., T. Gapotchenko, J. C. Varekamp, E. L. Mecray, and M. R. B. ten Brink. 2000. Benthic foraminifera and environmental changes in Long Island Sound. Journal of Coastal Research **16**:641–655.

Thorsen, T. A., and B. Dale. 1997. Dinoflagellate cysts as indicators of pollution and past climate in a Norwegian fjord. Holocene **7**:433–446.

Tikkanen, M., A. Korhola, H. Seppä, and J. Virkanen. 1997. A long-term record of human impacts on an urban ecosystem in the sediments of Töölönlahti Bay in Helsinki, Finland. Environmental Conservation **24**:326–337.

Toyoda, K., and H. Kitazato. 1995. Paleoenvironmental changes of Yokohama Port since 1870 based on benthic foraminiferal fossils. Changes in marine organisms and environments at Yokohama Port: research on fossils in sediment core samples, Yokohama Environmental Research Institute, Report no. 116:11–26.

Tsujimoto, A., R. Nomura, M. Yasuhara, H. Yamazaki, and S. Yoshikawa. 2006a. Impact of eutrophication on shallow marine benthic foraminifers over the last 150 years in Osaka Bay, Japan. Marine Micropaleontology **60**:258–268.

Tsujimoto, A., R. Nomura, M. Yasuhara, and S. Yoshikawa. 2006b. Benthic foraminiferal assemblages in Osaka Bay, southwestern Japan: faunal changes over the last 50 years. Paleontological Research **10**:141–161.

Tsujimoto, A., M. Yasuhara, R. Nomura, H. Yamazaki, Y. Sampei, K. Hirose, and S. Yoshikawa. 2008a. Development of modern benthic ecosystems in eutrophic coastal oceans: the foraminiferal record over the last 200 years, Osaka Bay, Japan. Marine Micropaleontology **69**:225–239.

Tsujimoto, A., M. Yasuhara, H. Yamazaki, K. Hirose, and S. Yoshikawa. 2008b. Environmental changes during the last 150 years in Osaka Bay, Japan: historical record of eutrophication based on microfossil assemblages. The Quaternary Research (Daiyonki-kenkyu) **47**:273–285.

Tuovinen, N., K. Weckström, and J. J. Virtasalo. 2010. Assessment of recent eutrophication and climate influence in the Archipelago Sea based on the subfossil diatom record. Journal of Paleolimnology **44**:95–108.

Wang, Z. H., K. Matsuoka, Y. Z. Qi, J. F. Chen, and S. H. Lu. 2004. Dinoflagellate cyst records in recent sediments from Daya Bay, South China Sea. Phycological Research **52**:396–407.

Weckström, K. 2006. Assessing recent eutrophication in coastal waters of the Gulf of Finland (Baltic Sea) using subfossil diatoms. Journal of Paleolimnology **35**:571–592.

Weckstrom, K., S. Juggins, and A. Korhola. 2004. Quantifying background nutrient concentrations in coastal waters: A case study from an urban embayment of the Baltic Sea. AMBIO **33**:324–327.

Weckström, K., A. Korhola, and J. Weckstrom. 2007. Impacts of eutrophication on diatom life forms and species richness in　coastal waters of the Baltic Sea. AMBIO **36**:155–160.

Willard, D. a., and T. M. Cronin. 2007. Paleoecology and ecosystem restoration: case studies from Chesapeake Bay and the Florida Everglades. Frontiers in Ecology and the Environment **5**:491-498.

Willard, D. A., T. M. Cronin, and S. Verardo. 2003. Late-Holocene climate and ecosystem history from Chesapeake Bay sediment cores, USA. Holocene **13**:201–214.

Witak, M., K. Boryn, and A. Mayer. 2005. Holocene environmental changes recorded by diatom stratigraphy in the Vistula Lagoon. Oceanological and Hydrobiological Studies **34**:111–133.

Witak, M., and D. Jankowska. 2005. The Vistula Lagoon evolution based on diatom records. Baltica **18**:68–76.

Witak, M., D. Jankowska, and H. Piekarek-Jankowska. 2006. Holocene diatom biostratigraphy of the SW Gulf of Gdańsk, Southern Baltic Sea (part I). Oceanological and Hydrobiological Studies **35**:307–329.

Witkowski, A., and J. Pampkowiak. 1995. Reconstructing the development of human impact from diatoms and ^210^Pb sediment dating (the Gulf of Gdansk-southern Baltic Sea). Geographia Polonica **65**:63–78.

Yasuhara, M., and H. Yamazaki. 2005. The impact of 150 years of anthropogenic pollution on the shallow marine ostracode fauna, Osaka Bay, Japan. Marine Micropaleontology **55**:63-74.

Yasuhara, M., H. Yamazaki, T. Irizuki, and S. Yoshikawa. 2003. Temporal changes of ostracode assemblages and anthropogenic pollution during the last 100 years, in sediment cores from Hiroshima Bay, Japan. Holocene **13**:527–536.

Yasuhara, M., H. Yamazaki, A. Tsujimoto, and K. Hirose. 2007. The effect of long-term spatiotemporal variations in urbanization-induced eutrophication on a benthic ecosystem, Osaka Bay, Japan. Limnology and Oceanography **52**:1633–1644.

Yokose, H., N. Momoshima, K. Matsuoka, Y. Hase, and E. Honza. 2005. Environmental assessments of Ariake Bay during the past 100 years based on marine sediments. Journal of Geography **114**:1–20.
